# Supplementary material for: H3BERTa: A CDR-H3-specific language model for antibody repertoire analysis
Source: Patterns (N Y). 2026 May 20;7(7):101561. doi: 10.1016/j.patter.2026.101561 (PMC13366522; doi:10.1016/j.patter.2026.101561)
Supplement: Document S1. Figures S1–S14 and Tables S1–S4 [file mmc1.pdf]

Patterns, Volume *7*

Supplemental information

H3BERTa: A CDR-H3-specific

language model for antibody repertoire analysis

Chiara Rodella and Thomas Lemmin

Supplementary Information

A

Isotype	n seq.	Mean MLM	Perplexity
IgG	19	1.42	4.14
IgA	90	2.10	8.15
IgE	53	1.38	3.98
IgM	54	1.67	5.30
IgD	46	1.45	4.27

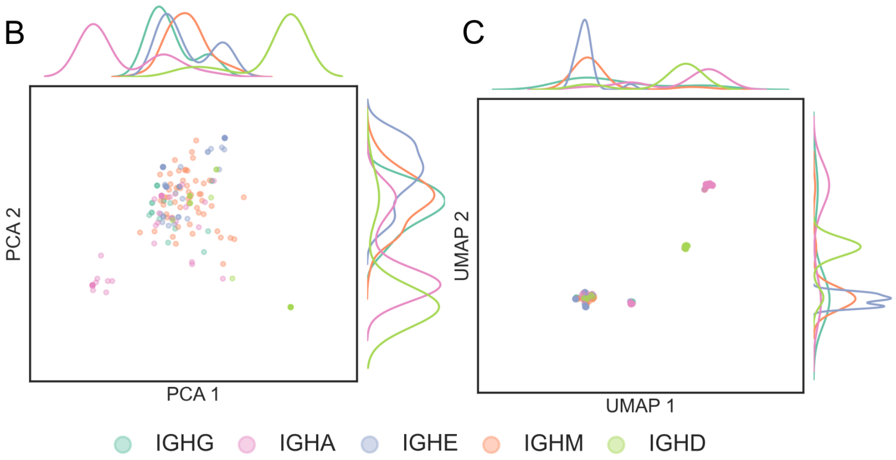

Figure S1: **H3BERTa performance and embedding structure across antibody isotypes.** (A) Mean masked language modeling (MLM) loss and perplexity for CDR-H3 sequences stratified by antibody isotype. (B) Principal component analysis (PCA) and (C) uniform manifold approximation and projection (UMAP) of H3BERTa-derived CDR-H3 embeddings, colored by isotype: IgG (light blue), IgA (pink), IgE (dark blue), IgM (red), and IgD (green). Each point represents a unique heavy-chain CDR-H3 sequence.

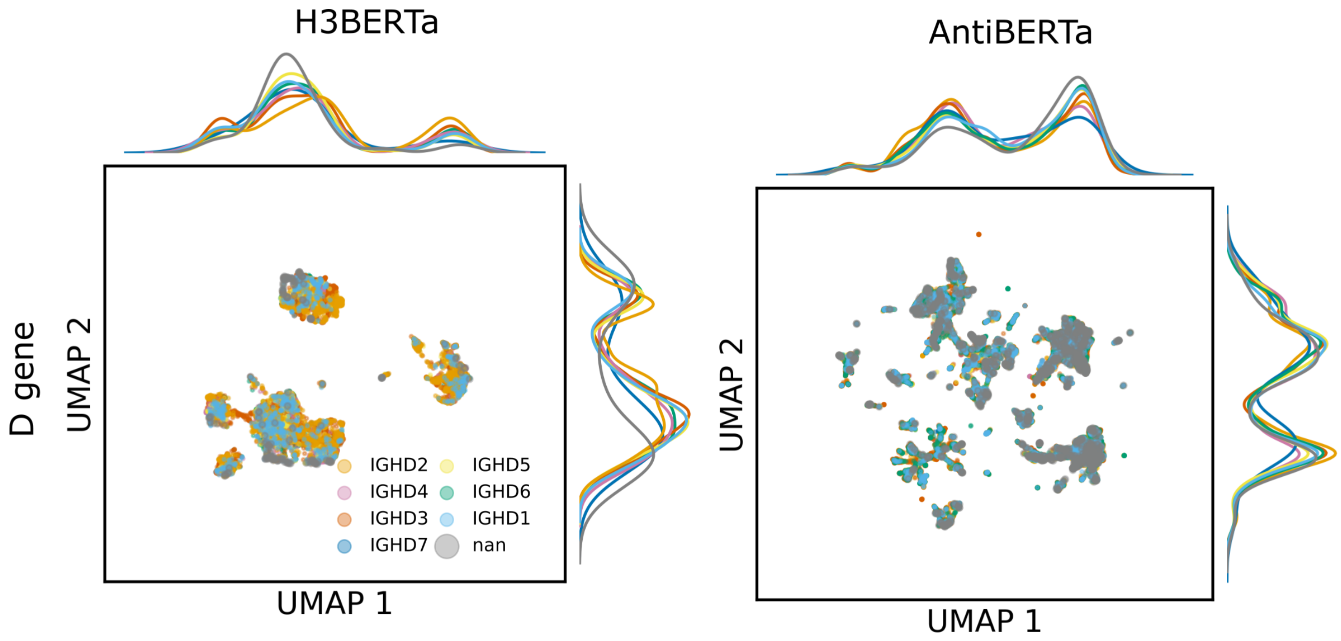

Figure S2: **UMAP visualization of H3BERTa and AntiBERTa-derived embeddings for CDR-H3 loops.** Each point represents a single CDR-H3 embedding colored by IGHD gene-segment usage. Marginal ridge plots alongside each UMAP axis depict the density of individual D-gene families.

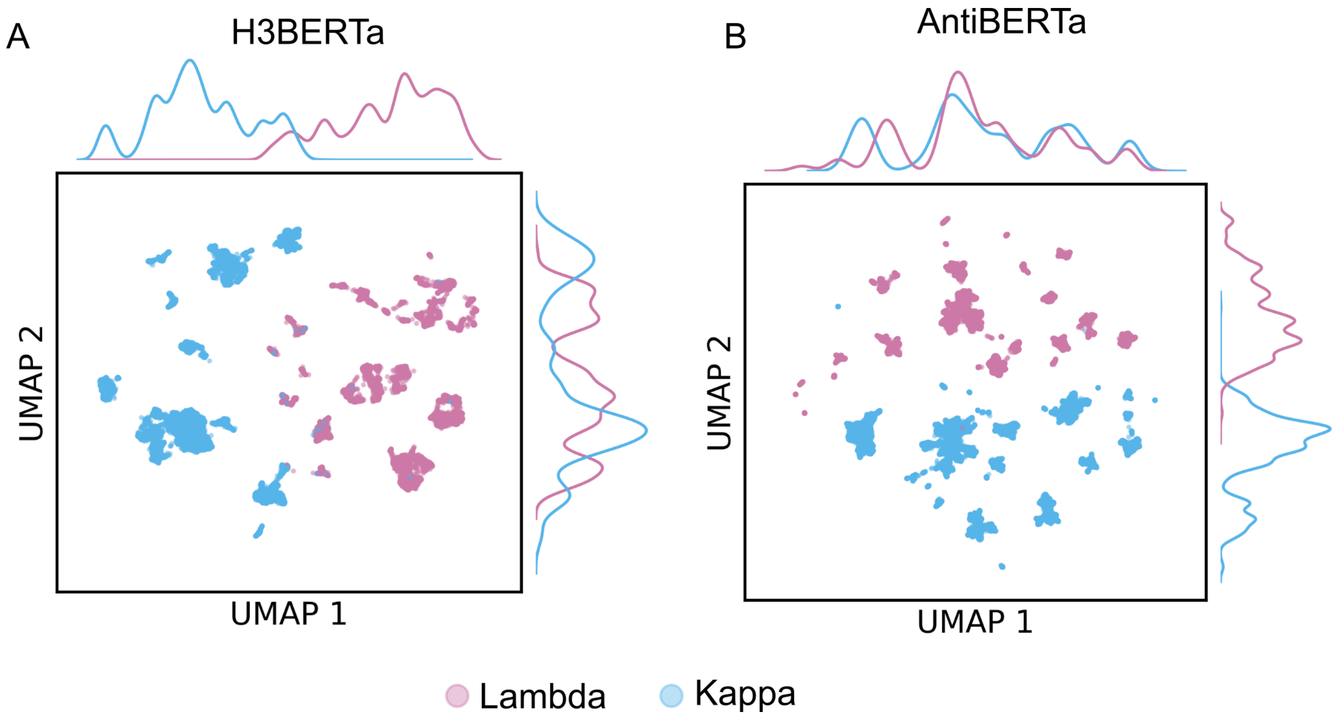

Figure S3: **Visualization of light-chain CDR3 embeddings.** Uniform manifold approximation and projection (UMAP) of CDR-L3 embeddings generated by H3BERTa (A) and full-length embeddings generated by AntiBERTa2 (B). Each point represents a unique antibody sequence and is colored by light-chain locus: lambda (pink) and kappa (blue).

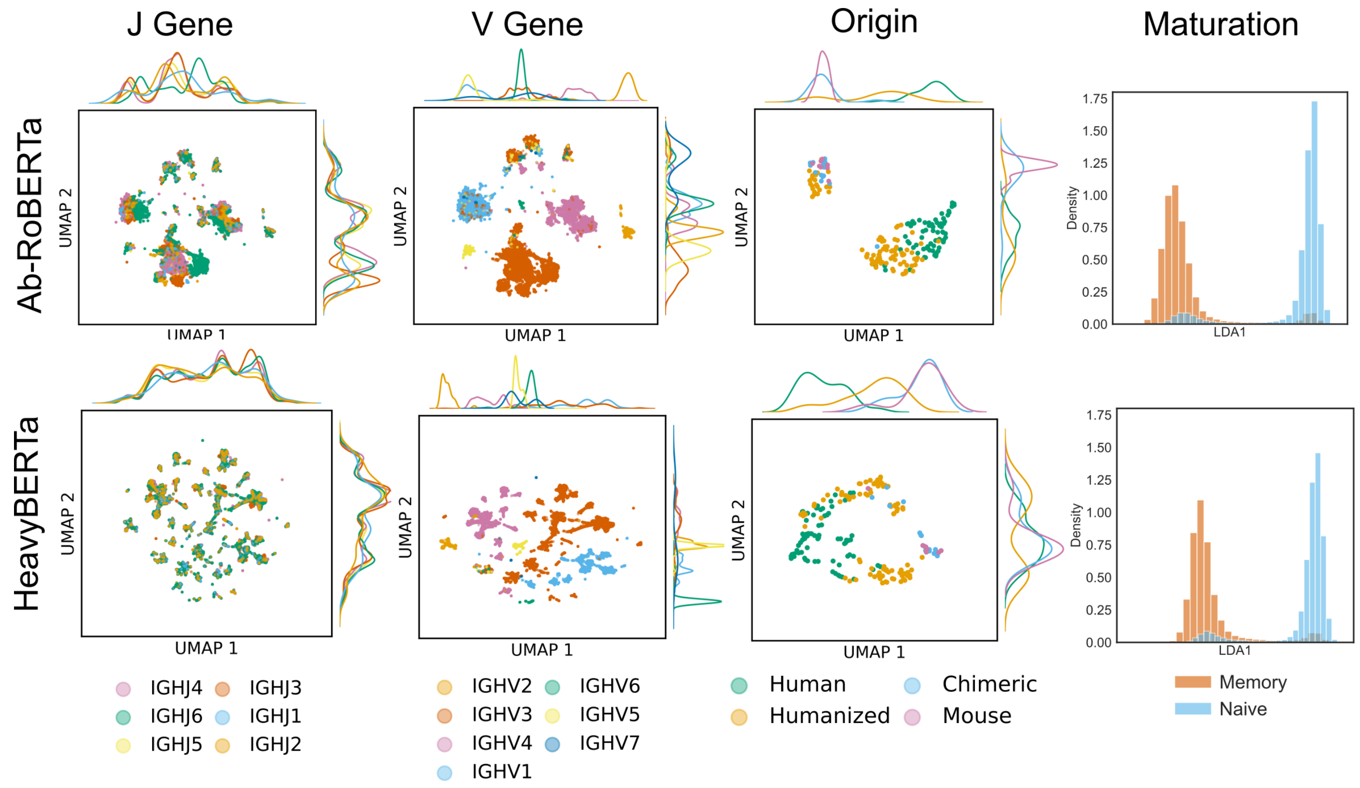

Figure S4: **Sequence embeddings generated by Ab-RoBERTa (top row) and HeavyBERTa (bottom row), visualized using UMAP or Linear Discriminant Analysis (LDA).** For UMAP panels, marginal distributions along each axis are shown as kernel density ridges. Each point represents a unique antibody heavy-chain sequence. UMAP projection of embeddings, colored by (A) IGHJ gene segment and (B) IGHV gene segment. (C) UMAP projection of embeddings from therapeutic antibodies, colored by annotated species origin: Human (green), Humanized (orange), Chimeric (light blue), and Mouse (pink). (D) LDA projection of embeddings from naive (blue) and memory (orange) B-cell sequences.

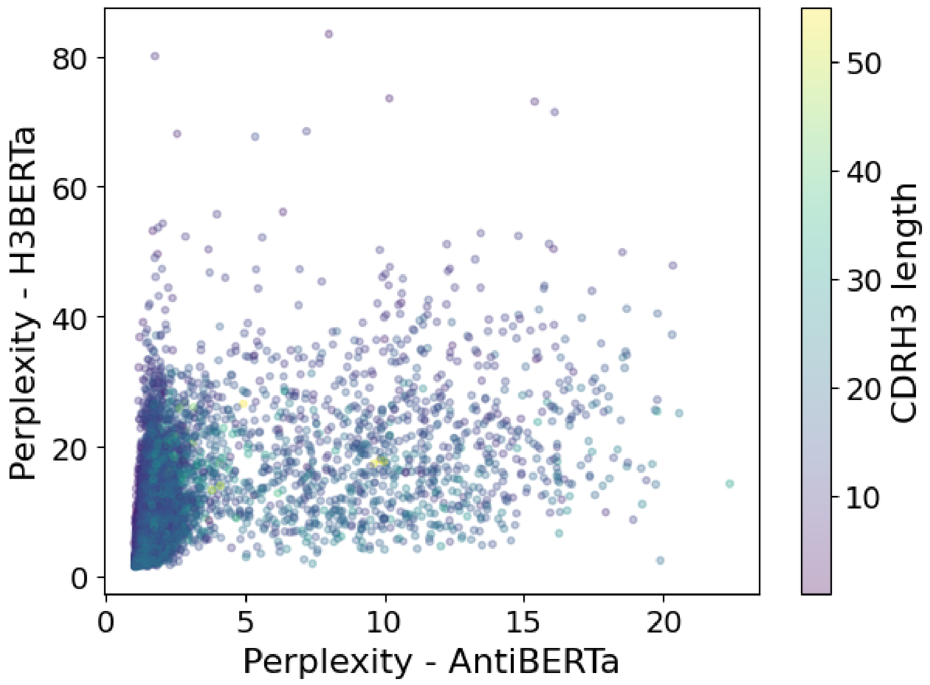

Figure S5: **Comparison of perplexity values calculated with AntiBERTa2 (x-axis) and H3BERTa (y-axis) on a healthy repertoire, specifically on the heavy chain and CDR-H3 loop, respectively.** Each point represents a unique CDR-H3 sequence: shifts to the right indicate an increase in perplexity for AntiBERTa2, and shifts upwards indicate an increase for H3BERTa. Points are colored according to CDR-H3 length using a continuous colormap.

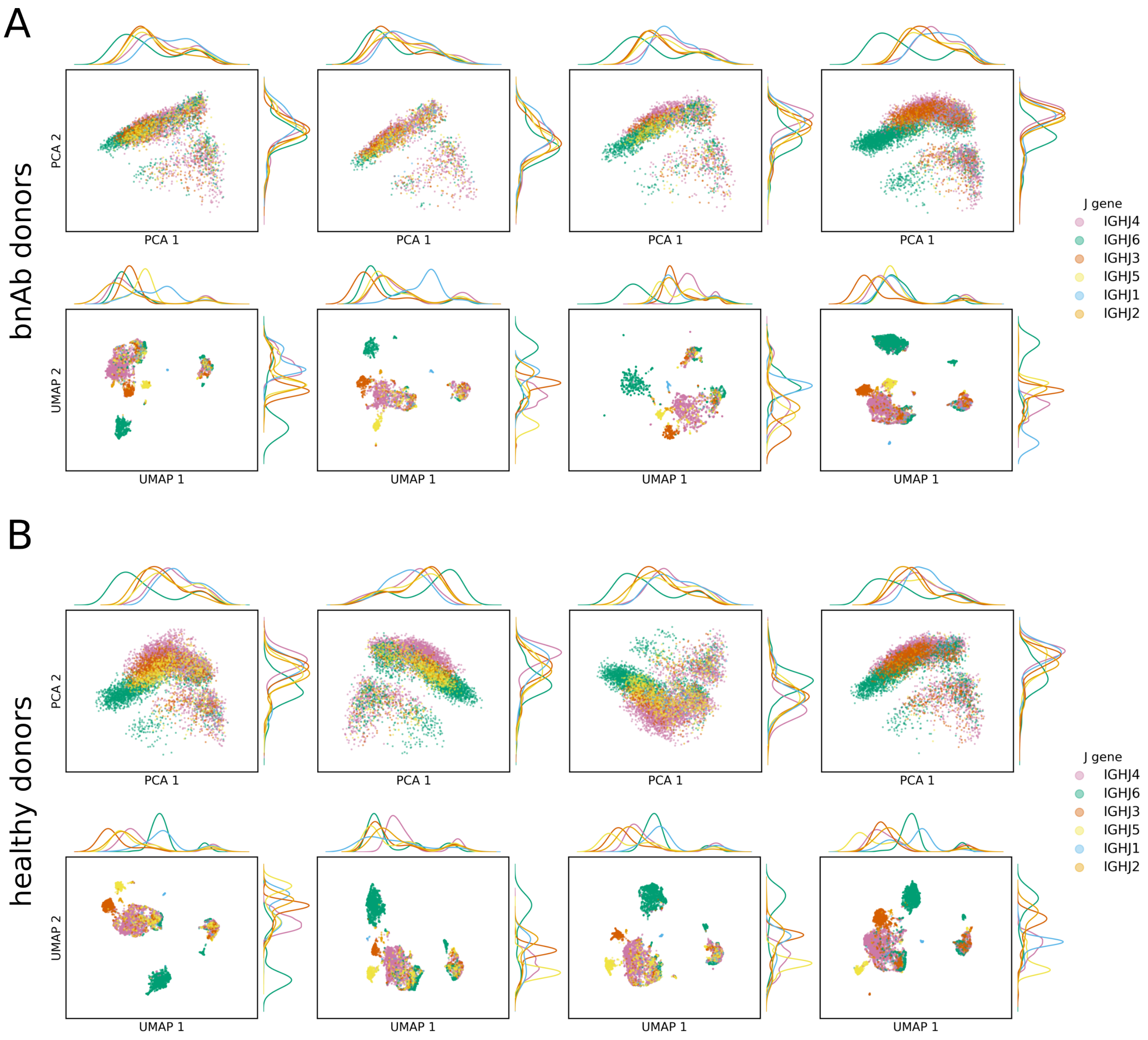

Figure S6: **Visualization of H3BERTa-derived embeddings for CDR-H3 loops from (A) bnAb donors and (B) healthy donors.** Each point represents a single CDR-H3 embedding colored by by IGHJ gene-segment usage. Marginal ridge plots alongside each PCA and UMAP axis depict the density of individual J-gene families.

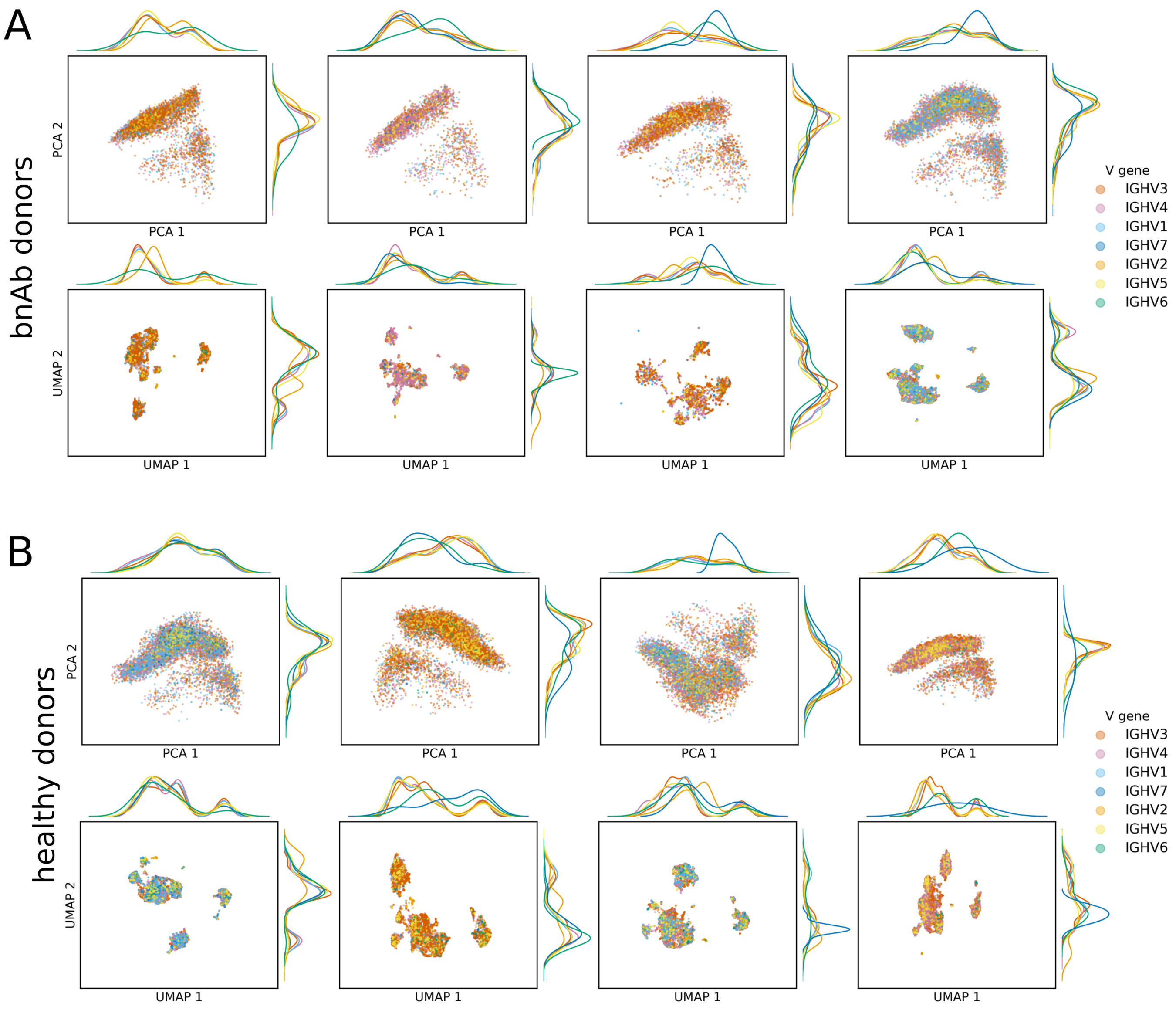

Figure S7: **Visualization of H3BERTa-derived embeddings for CDR-H3 loops from (A) bnAb donors and (B) healthy donors.** Each point represents a single CDR-H3 embedding colored by by IGHV gene-segment usage. Marginal ridge plots alongside each PCA and UMAP axis depict the density of individual V-gene families.

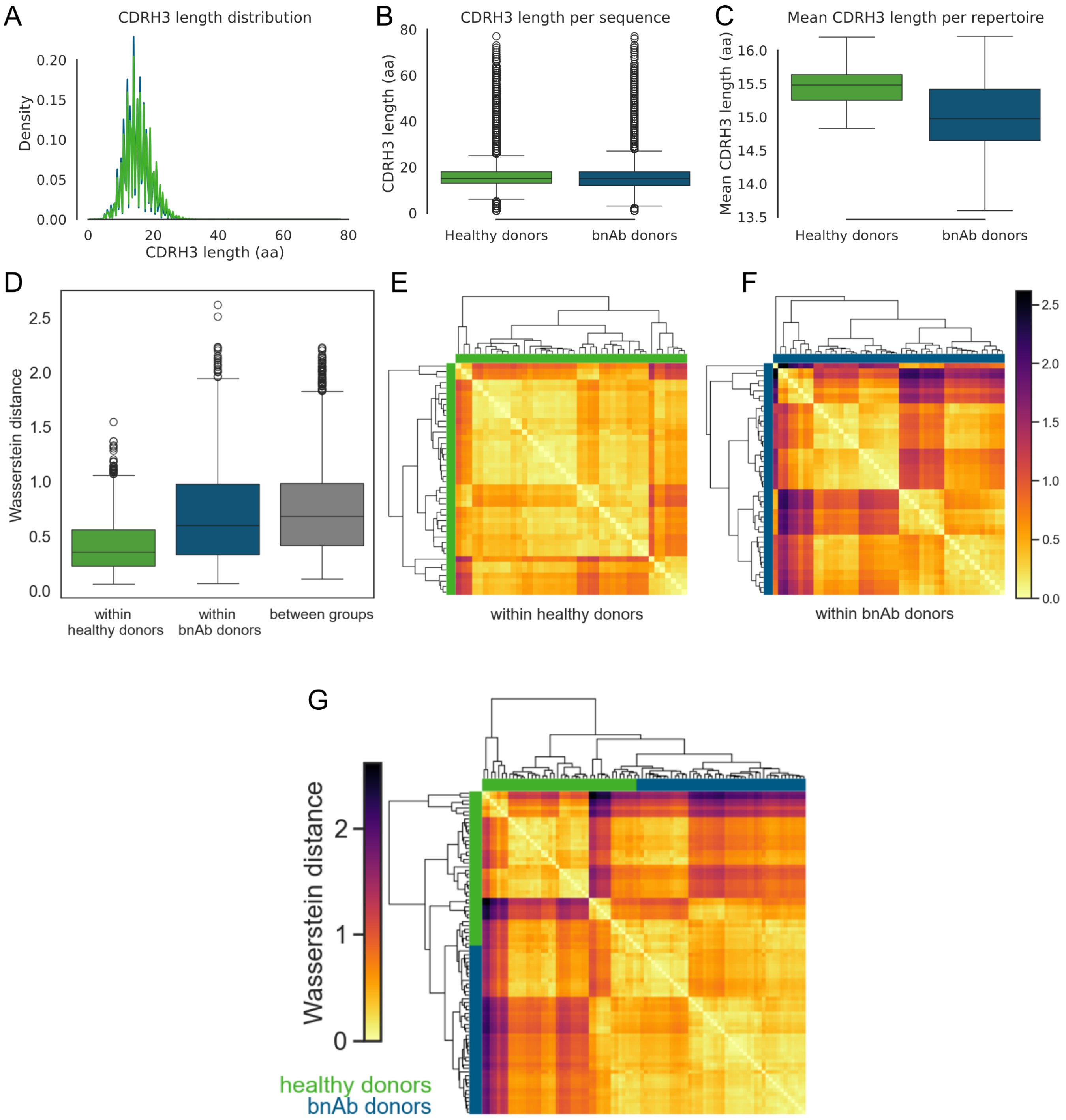

Figure S8: **Characterization of CDR-H3 length distributions.** (A) Kernel density estimate of CDR-H3 length distributions for healthy donors (green) and bnAb patients (blue). (B) Box plot of individual CDR-H3 sequence lengths; boxes indicate mean ± standard deviation, and outliers are shown as individual points. (C) Box plot of the average CDR-H3 length per repertoire for healthy and bnAb donors. (D) Box plot of Wasserstein distances between CDR-H3 length distributions across repertoires. (E-G) Ward clustering based on Wasserstein distances of CDR-H3 length distributions: (E) healthy donors, (F) bnAb donors, and (G) combined healthy and bnAb donors.

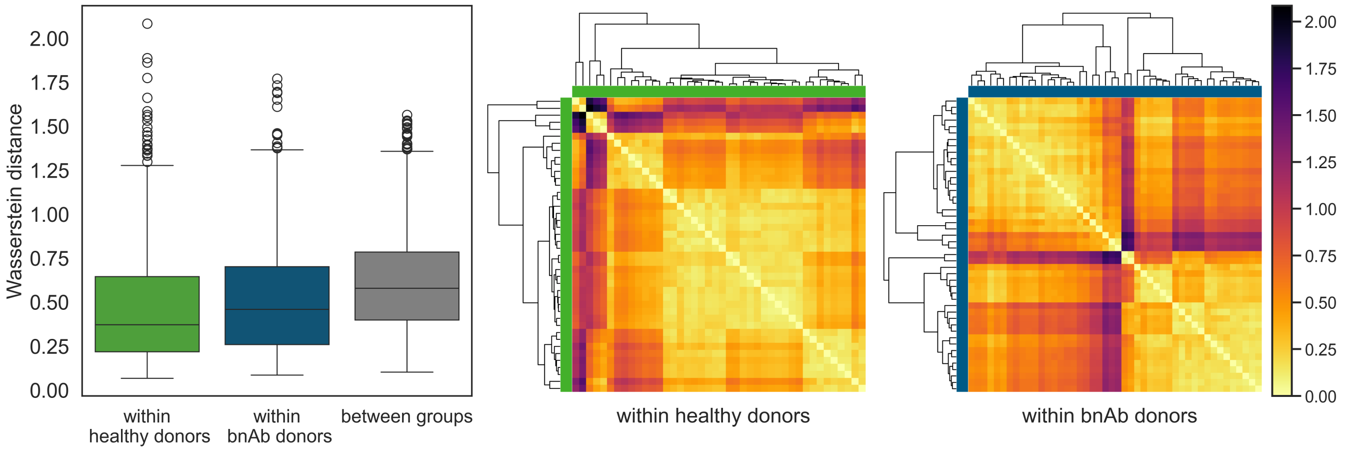

Figure S9: **Wasserstein distances analysis**(A) Box plots of pairwise Wasserstein distances between CDR-H3 perplexity distributions from H3BERTA. Distances are shown for comparisons within healthy donors (green), within bNAb donors (blue), and between the two cohorts (grey). Lower values indicate more similar repertoires. Healthy donors display the lowest median distance, whereas inter-cohort comparisons are shifted upward. (B) Heatmaps of the same pairwise distances. The left panel includes only healthy donors; the right panel includes only bNAb donors. Samples are ordered by hierarchical clustering to highlight similarity patterns. Colours progress from yellow (0; high similarity) through orange to black (= 2; high dissimilarity).

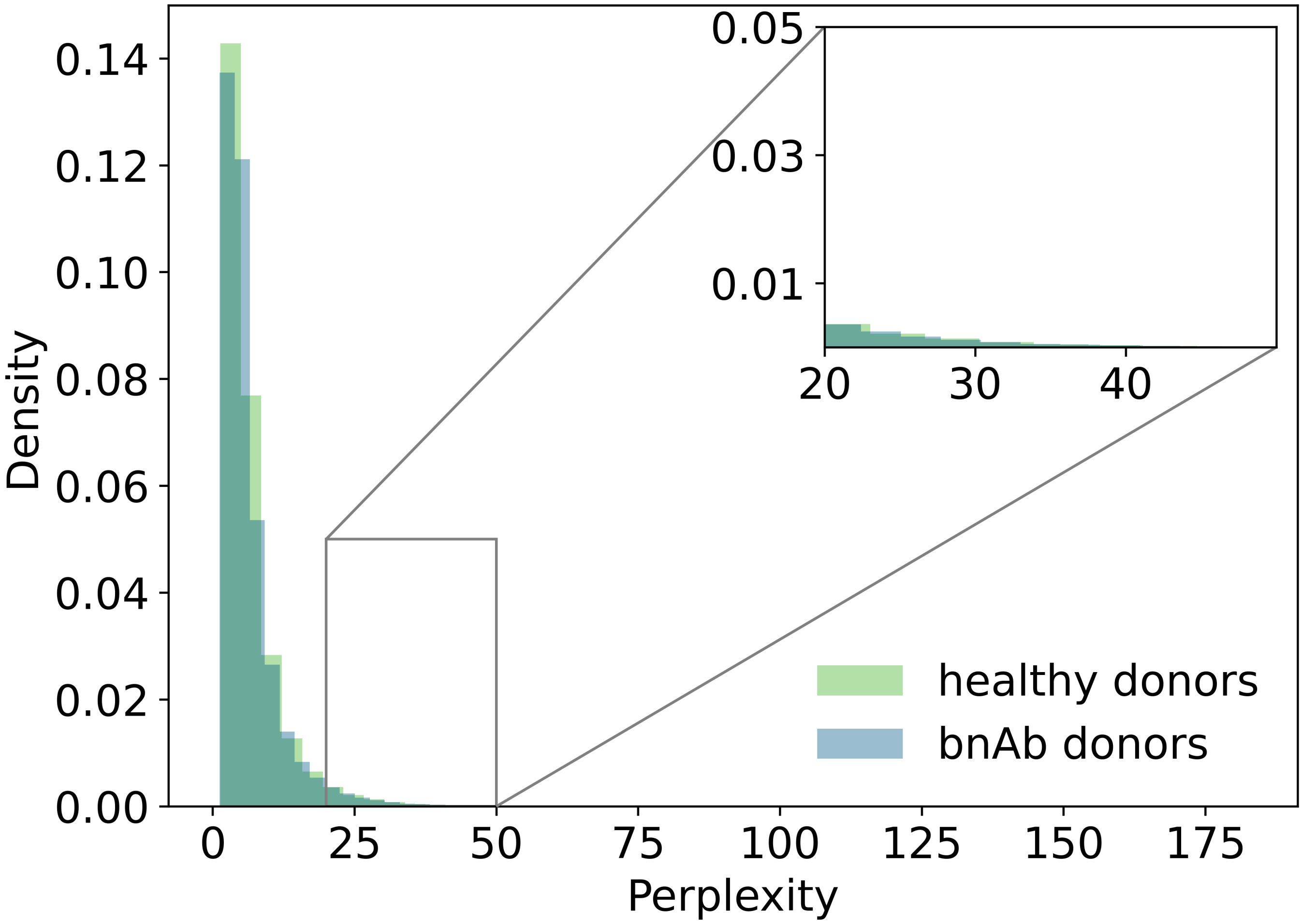

Figure S10: **Perplexity distributions of CDR-H3 sequences from healthy and bnAb donors.** Kernel density estimates show that both distributions are heavily right-skewed, with most sequences having low perplexity scores. An inset highlights the right tail of the distribution, where rare high-perplexity sequences are more visible.

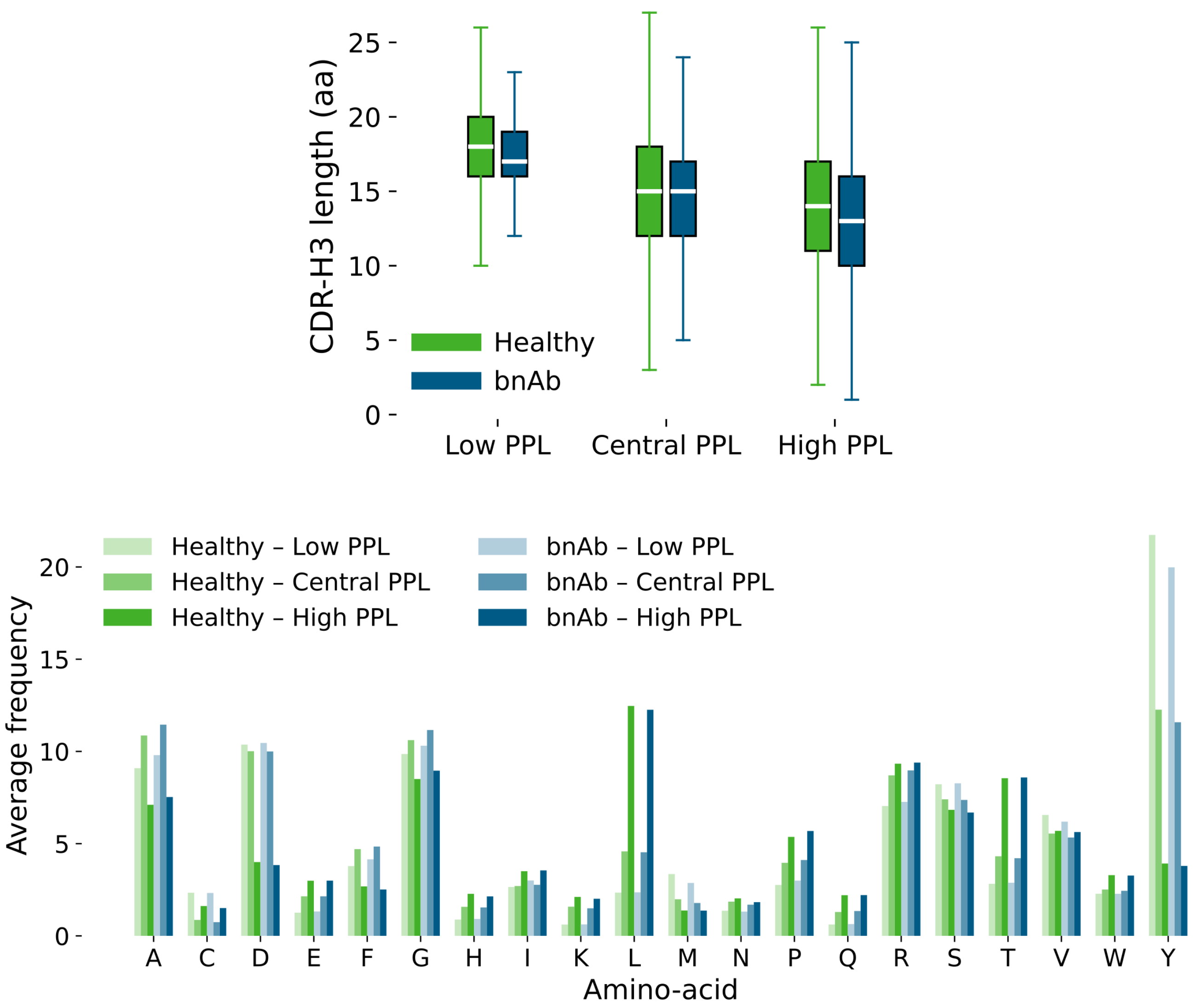

Figure S11: **Sequence features of CDR-H3 stratified by pseudo-perplexity (PPL) in healthy and bnAb donors.** Top: Box plots of CDR-H3 lengths for low-, central-, and high-PPL sequences, shown separately for healthy donors (green) and bnAb donors (blue). Bottom: Mean amino acid frequencies within each PPL bin, displayed for healthy donors (shades of green) and bnAb donors (shades of blue).

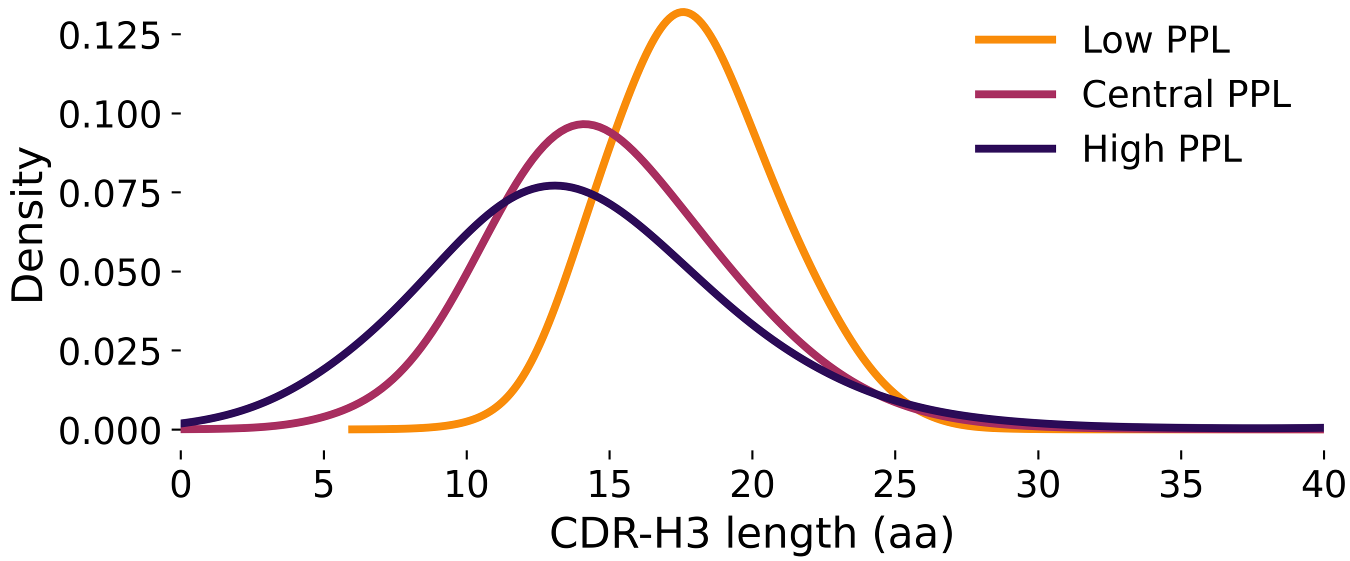

Figure S12: **Length distribution of CDR-H3 sequences across perplexity-defined groups.** Kernel density estimates of CDR-H3 amino acid lengths for sequences categorized into Low PPL (orange), Central PPL (magenta), and High PPL (dark purple) groups. While all groups display a peak in the 12-18 amino acid range, Low PPL sequences tend to be longer, indicating a potential association between lower model perplexity and naive CDR-H3 regions. CDR-H3 sequences with lengths greater than 40 amino acids were removed from the plot as outliers to improve the clarity of the density distribution.

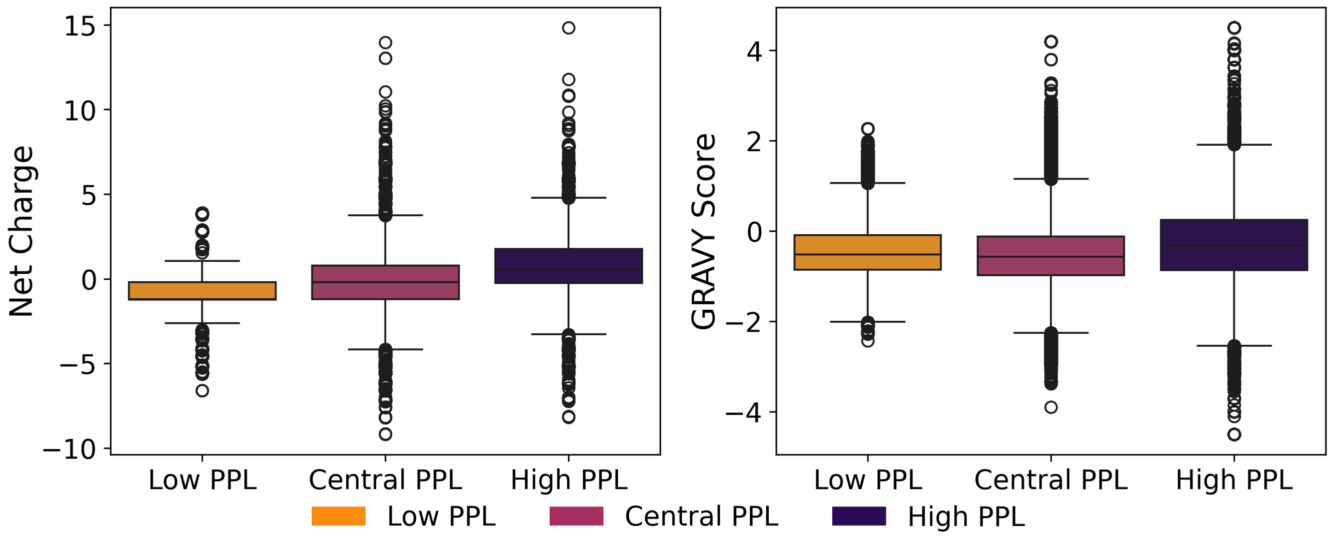

Figure S13: **Biochemical properties of CDR-H3 sequences across perplexity-defined groups.** Boxplots of (A) net charge at physiological pH (7.0) and (B) hydrophobicity (GRAVY score) for CDR-H3 sequences stratified by model-derived perplexity into Low, Central, and High PPL groups. Sequences in the Low PPL group exhibit significantly lower net charge compared to Central and High PPL groups (all $p < 0.001$, Mann-Whitney U test), suggesting a possible association between low perplexity and reduced electrostatic diversity. In contrast, GRAVY scores are relatively stable across groups, with only minor shifts despite statistically significant differences, indicating that overall hydrophobicity is not strongly differentiated by perplexity. Color-coded legends indicate PPL groups.

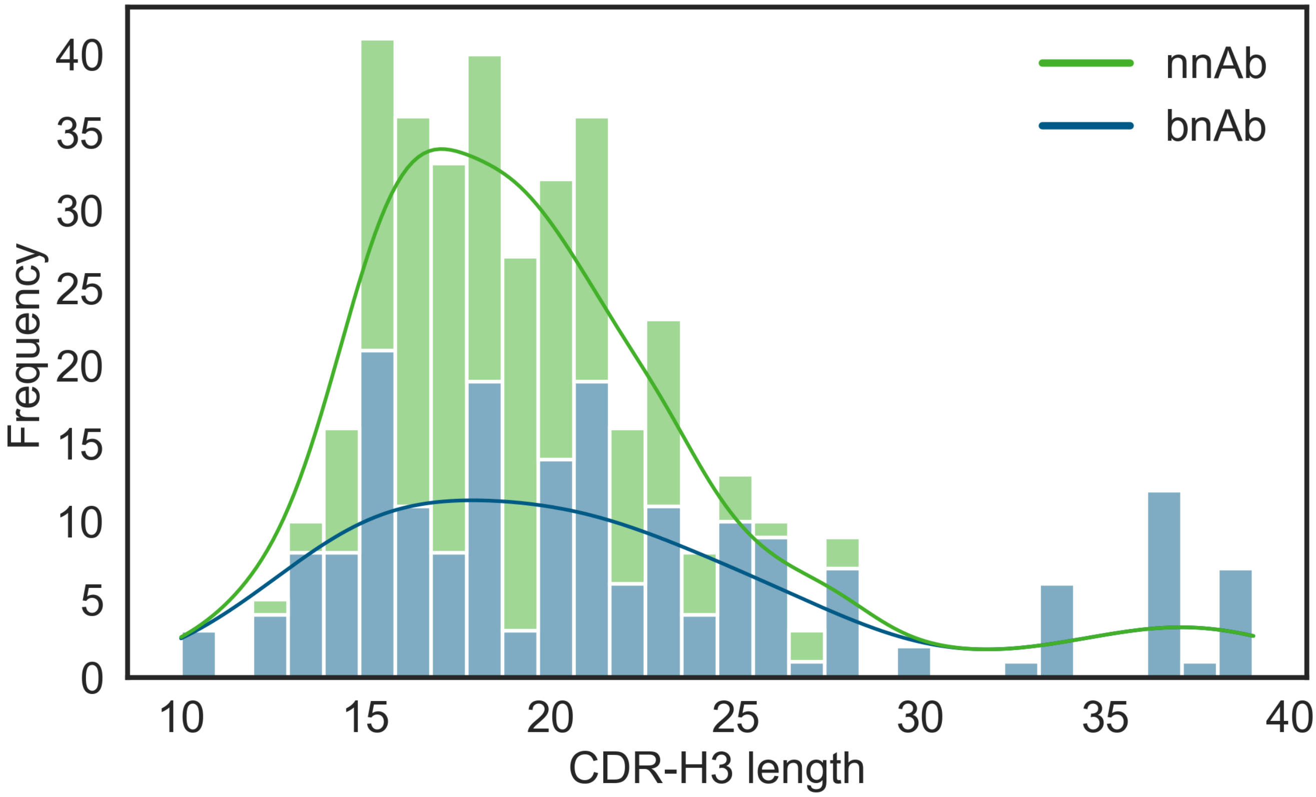

Figure S14: **Length distribution of CDR-H3 loops in bnAb and nnAb repertoires.** Histogram and kernel density estimates (KDE) of CDR-H3 amino acid lengths for broadly neutralizing antibodies (bnAbs, blue) and non-neutralizing antibodies (nnAbs, green). While both distributions peak in the 14-18 residue range, bnAbs tend to exhibit a broader and slightly right-shifted distribution, with a higher proportion of longer CDR-H3 loops compared to nnAbs.

Table S1: Mann–Whitney U test results for Net Charge at pH 7 and Hydrophobicity (GRAVY) across PPL groups.

**Comparison**	**Property**	**U statistic**	**p-value**
Low PPL vs Central PPL	Net Charge @ pH 7	1.26×10^{12}	$p < 0.001$
	Hydrophobicity (GRAVY)	1.69×10^{12}	$p < 0.001$
Low PPL vs High PPL	Net Charge @ pH 7	4.71×10^{10}	$p < 0.001$
	Hydrophobicity (GRAVY)	5.09×10^{10}	$p < 0.001$
Central PPL vs High PPL	Net Charge @ pH 7	1.16×10^{12}	$p < 0.001$
	Hydrophobicity (GRAVY)	8.12×10^{11}	$p < 0.001$

Table S2: SVM performance on validation and test sets. Metrics are reported overall and per class. Acc. = Accuracy, P = Precision, R = Recall, F1 = F1-score.

**Set**	**Acc.**	**bnAbs**			**nnAbs**		
		P	R	F1	P	R	F1
Validation	92.0%	1.00	0.83	0.91	0.86	1.00	0.92
Test	93.0%	0.95	0.90	0.92	0.90	0.95	0.93

Table S3: GAN-H3BERTa performance on validation and test sets. Metrics are reported overall and per class. Acc. = Accuracy, MCC = Matthews correlation coefficient, AUC = Area under the ROC curve, P = Precision, R = Recall, F1 = F1-score.

**Set**	**Acc.**	**MCC**	**AUC**	**bnAbs**			**nnAbs**		
				**P**	**R**	**F1**	**P**	**R**	**F1**
Validation	83.3%	0.67	0.83	0.80	0.89	0.84	0.88	0.78	0.82
Test	85.0%	0.71	0.85	0.79	0.95	0.86	0.94	0.75	0.83

Table S4: GAN-H3BERTa seed sensitivity analysis. Metrics are reported per seed on validation (val) and test sets, as computed with sklearn macro averages.

**Seed**	**Val Accuracy**	**Val F1**	**Test Accuracy**	**Test F1**
Model 1	0.81	0.80	0.82	0.82
Model 2	0.94	0.94	0.82	0.82
Model 3	0.81	0.80	0.85	0.85
Mean	0.85	0.85	0.83	0.83
Std	0.06	0.07	0.02	0.02
