# Supplementary material for: H3BERTa: A CDR-H3-specific language model for antibody repertoire analysis
Source: Patterns (N Y). 2026 May 20;7(7):101561. doi: 10.1016/j.patter.2026.101561 (PMC13366522; doi:10.1016/j.patter.2026.101561)
Supplement: Document S2. Article plus supplemental information [file mmc2.pdf]

Article

Patterns

H3BERTa: A CDR-H3-specific language model for antibody repertoire analysis

Graphical abstract

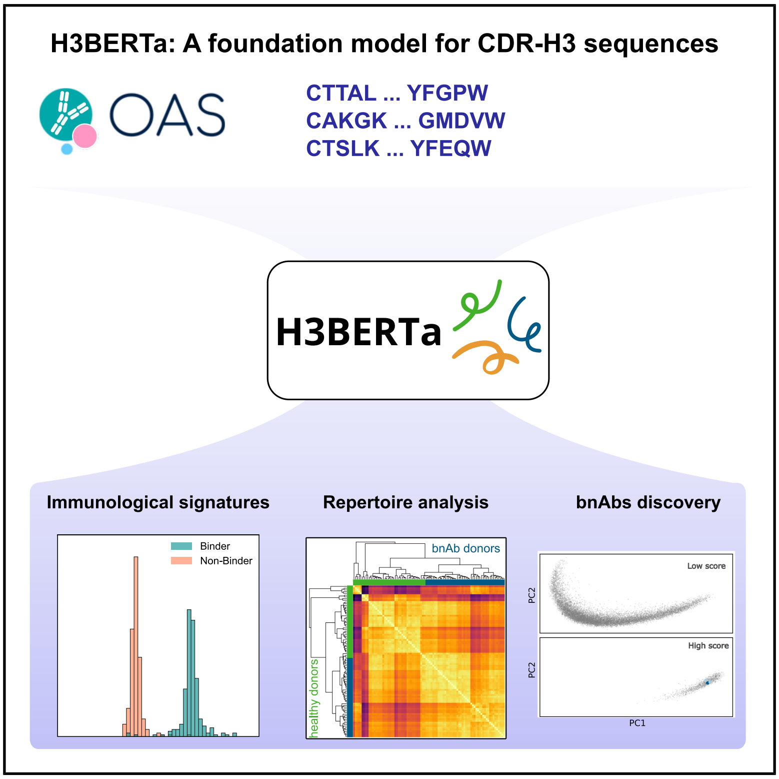

Highlights

- A language model trained only on antibody segment CDR-H3 captures immune signals
- Short, highly variable sequence regions encode rich information on antibody function
- Pseudo-perplexity profiles can identify differences between immune states
- This short-segment approach enables antibody discovery from limited labeled data

Authors

Chiara Rodella, Thomas Lemmin

Correspondence

thomas.lemmin@unibe.ch

In brief

Identifying functional antibodies from vast sequencing datasets remains a major challenge. This study shows that focusing on a short, highly variable region of antibodies is sufficient to capture key biological signals. A compact language model (H3BERTa) developed and trained only on this region enables efficient analysis of immune repertoires, distinguishes health and disease states, and supports antibody discovery with limited data. The work highlights how targeted modeling of informative sequence regions can improve scalability and interpretability in computational biology.

Check for updates

Rodella & Lemmin, 2026, Patterns 7, 101561
July 10, 2026 © 2026 The Author(s). Published by Elsevier Inc.
https://doi.org/10.1016/j.patter.2026.101561

CellPress

Patterns

CellPress
OPEN ACCESS

Article

H3BERTa: A CDR-H3-specific language model for antibody repertoire analysis

Chiara Rodella[1,2] and Thomas Lemmin[1,3,*]
[1]Institute of Biochemistry and Molecular Medicine (IBMM), University of Bern, Bühlstrasse 28, 3012 Bern, Switzerland
[2]Graduate School for Cellular and Biomedical Sciences (GCB), University of Bern, Mittelstrasse 43, 3012 Bern, Switzerland
[3]Lead contact
*Correspondence: thomas.lemmin@unibe.ch
https://doi.org/10.1016/j.patter.2026.101561

THE BIGGER PICTURE The immune system generates an enormous diversity of antibodies to recognize and neutralize pathogens. Advances in DNA sequencing now allow researchers to read millions of antibody sequences at once, offering unprecedented insight into immune responses. However, identifying which of these sequences are functionally relevant, such as those that bind pathogens or could serve as therapeutics, remains a major challenge. Current computational approaches often analyze full antibody sequences; this, unfortunately, can dilute important signals and requires large amounts of labeled data that are rarely available.

In this study, we explore a more focused strategy: instead of modeling the entire antibody, we investigate whether its most variable and functionally critical segment alone can capture meaningful biological information. To this aim, we developed and trained a language model (H3BERTa) exclusively on this short-segment region and demonstrated that it indeed retains rich information related to immune function, disease state, and antibody activity. This simplified representation enables an efficient analysis of large antibody datasets and supports downstream tasks such as identifying promising therapeutic candidates, even when labeled data are scarce. It can be expected that this approach could help make immune repertoire analysis more scalable and accessible, particularly in settings where data are limited or heterogeneous. More broadly, it illustrates how narrowing the scope of machine learning models to the most informative regions of biological sequences can improve interpretability and performance. Such strategies may extend beyond immunology to other areas of biology where identifying key functional signals within complex, high-dimensional data remains a central challenge.

SUMMARY

Antibodies are central to immune defense and therapeutic design, yet predicting functional sequences remains challenging. Deep learning models trained on full variable regions often struggle due to sparse experimental data, signal dilution from conserved framework residues, and extreme diversity of hypervariable loops. The heavy-chain complementarity-determining region 3 (CDR-H3) is the most variable segment, shaping antigen specificity and immune diversity. Here, we present H3BERTa, a language model trained solely on CDR-H3 sequences to assess the extent of biological information encoded by this region. H3BERTa embeddings recapitulate immunologically relevant features, including J-gene usage, inferred B cell maturation state, and antigen binding. Pseudo-perplexity profiles enable repertoire analysis, distinguishing healthy from human immunodeficiency virus type 1 (HIV-1)-derived sequences and suggesting measurable immune response signatures. These embeddings support classifiers for broadly neutralizing antibodies using limited labeled data, highlighting utility for antibody discovery. CDR-H3 alone encodes a rich immunological signal, which H3BERTa captures, offering a focused tool for repertoire analysis and antibody engineering.

This is an open access article under the CC BY license (http://creativecommons.org/licenses/by/4.0/).

INTRODUCTION

Modeling antigen specificity within the immense diversity of B cell receptor (BCR) repertoires remains a major computational challenge. Recent antibody language models have shown promise in learning structure-function relationships directly from sequences.[1–4] However, many of these approaches rely on full-length variable regions, which increases data requirements and computational costs and potentially obscures functionally relevant signals with conserved framework residues. These challenges underscore the need for more efficient computational methods capable of detecting functional antibody signatures using minimal sequence information. Thus, it is of interest to explore a more focused strategy centered on the immunoglobulin (Ig) heavy-chain complementarity-determining region 3 (CDR-H3), a short but highly variable segment that plays a central role in antigen recognition. CDR-H3 is known to contribute disproportionately to binding specificity and diversity and encodes signatures of immune selection, clonal expansion, and somatic mutation.[5] We therefore hypothesized that models trained solely on CDR-H3 could extract immunologically meaningful representations, particularly in settings where annotated data are sparse or full-length sequences are unavailable.

Broadly neutralizing antibodies (bnAbs) offer a compelling test case for evaluating such a strategy. Although immune repertoire sequencing can now generate millions of antibody sequences per donor,[4–6] only a small fraction exhibit high-affinity or broadly reactive properties.[7,8] bnAbs are of particular interest because they recognize conserved epitopes across diverse viral strains[9,10] and can neutralize challenging pathogens such as human immunodeficiency virus type 1 (HIV-1),[11,12] influenza,[10,13] and coronaviruses.[9] However, their extreme rarity in natural repertoires and the difficulty of linking sequence to function make their discovery both computationally and experimentally demanding.

Among bnAbs, those targeting HIV-1 are among the most extensively studied.[14] These antibodies neutralize a broad spectrum of HIV-1 strains by binding conserved regions of the viral envelope glycoprotein, such as the CD4-binding site, the V1/V2 apex, and the membrane-proximal external region.[11,12] HIV-1 bnAbs have provided key insights into B cell maturation, immune tolerance, and host-virus co-evolution,[12,15] yet they typically develop only in a minority of infected individuals,[8] following prolonged antigenic stimulation and extensive somatic hypermutation. Consequently, identifying them experimentally remains labor- and resource-intensive.[16]

In this study, we introduce H3BERTa, a transformer-based language model trained exclusively on CDR-H3 sequences. Despite its narrow input scope, H3BERTa learns biologically meaningful representations that capture features such as gene usage, B cell maturation state, and antigen binding. Leveraging these embeddings, we demonstrate the model's ability to distinguish repertoire-level differences between healthy individuals and patients infected with HIV and explore the potential for a classifier that uses CDR-H3 embeddings to identify candidate HIV-1 bnAbs. This CDR-H3-focused modeling approach enables repertoire-wide screening using only short sequence fragments and highlights a promising direction for accelerating antibody discovery, particularly in data-limited settings.

RESULTS

The Ig heavy-chain CDR-H3 is critical for antigen specificity and commonly used in repertoire analysis, often through methods that rely on simple features such as length or sequence similarity.[17,18] However, these approaches offer a limited view of the complex biological information encoded within this highly variable region. We hypothesized that, despite its short length, the CDR-H3 region encodes sufficient immunological information to support nuanced repertoire analysis using advanced deep learning techniques.

To investigate this hypothesis, we developed H3BERTa, a transformer-based language model that leverages the RoBERTa architecture[19] and comprises approximately 86 million trainable parameters. H3BERTa was pretrained on approximately 18 million CDR-H3 sequences obtained from the Observed Antibody Space (OAS) database.[20] This extensive dataset was specifically curated to include IgG and IgA isotypes from healthy, unvaccinated donors, thus providing a broad and biologically meaningful foundation reflecting major functional antibody classes in systemic and mucosal immunity (Figure 1A). IgG antibodies are typically the most abundant in serum and mediate a wide range of effector functions, whereas IgA antibodies play a crucial role at mucosal surfaces.[21]

Comparison of H3BERTa and full-length antibody language models

H3BERTa was trained for 113 epochs, converging with a final masked language modeling loss of 1.58. To assess whether H3BERTa generalizes across antibody isotypes, we evaluated masked language modeling performance on small sets of IgE,[22] IgM,[23] and IgD[24] CDR-H3 sequences drawn from independent studies. The mean loss and perplexity values were comparable to those observed for IgG and IgA, indicating consistent performance across diverse isotypes (Figure S1A). While linear projection via principal-component analysis (PCA) did not reveal clear separation between isotypes, nonlinear embedding visualization using uniform manifold approximation and projection (UMAP) showed distinct clustering, suggesting that H3BERTa captures isotype-specific features in a nonlinear fashion (Figures S1B and S1C). Together, these results support the robustness and broad applicability of H3BERTa across multiple antibody classes.

We then compared H3BERTa to AntiBERTa2,[25] a transformer-based model pretrained on full variable regions of Ig heavy chains. This comparison allowed us to evaluate how a minimal, CDR-H3-centric representation performs relative to models trained on more extensive sequence input. A UMAP visualization of H3BERTa embeddings revealed a repertoire organization primarily influenced by the J-gene segment (Figure 2A). This demonstrates H3BERTa's capacity to organize sequences based on intrinsic genomic signals associated with the CDR-H3 recombination process. In contrast, embeddings from AntiBERTa2 showed a stronger clustering by V-gene segment (Figure 2B), reflecting the model's access to the framework and other CDR regions, where V-gene identity is a dominant signal. No discernible clustering by D-gene usage was observed (Figure S2), consistent with the well-documented difficulty in accurately assigning D-gene segments due to their short length and high sequence

CellPress
OPEN ACCESS

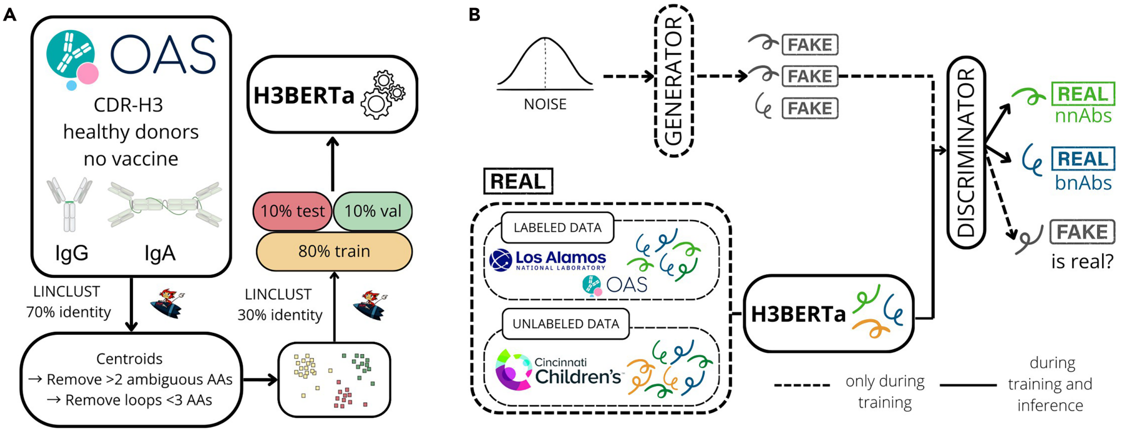

Figure 1. Overview of the H3BERTa deep learning framework and its application to broadly neutralizing antibody classification

(A) H3BERTa pretraining. Data preparation and training pipeline for H3BERTa used diverse CDR-H3 sequences from the Observed Antibody Space (OAS) database.

(B) Semi-supervised broadly neutralizing antibody (bnAb) classification with GAN-H3BERTa. The discriminator is jointly trained to distinguish real from synthetic embeddings and to classify sequences as broadly neutralizing (bnAb) or non-neutralizing (nnAb) antibodies. Post-training, the discriminator is used to predict bnAbs in antibody repertoires.

variability. To determine whether embedding extends beyond heavy-chain sequences, we performed an analogous analysis on light-chain CDR-L3 regions. Both H3BERTa and AntiBERTa2 embeddings showed clear separation of kappa and lambda chains, indicating that locus-specific signatures are robustly encoded despite differences in input sequence chain (Figure S3).

Next, we evaluated each model's ability to capture species-specific features in therapeutic antibody sequences by generating embeddings for antibodies from the Thera-SAbDab database,[26] which includes approved and clinical-stage antibodies annotated by species of origin. AntiBERTa2 embeddings exhibited clear clustering by species (Figure 2C), reflecting the presence of species-distinctive sequence features in the variable domain. These include framework residues that are less frequently mutated during affinity maturation and thus preserve phylogenetic and germline-derived differences between species and antibody formats. Since AntiBERTa2 processes the entire variable region, it captures these invariant residues and species-specific motifs that distinguish engineered from native antibodies. In contrast, H3BERTa embeddings showed a more diffuse distribution, with only partial separation for some human antibodies. This could suggest that weak species-related signals are still partially encoded within the CDR-H3 region.

In addition to gene-specific signals, we sought to determine whether the learned embeddings capture biologically meaningful functional and developmental properties of antibody sequences. We first evaluated whether H3BERTa embeddings encode B cell maturation state, a key dimension of repertoire evolution. Since UMAPs were largely dominated by gene segment usage, we applied linear discriminant analysis (LDA) to isolate features associated with maturation state (Figure 3). When applied to labeled naive and memory B cell sequences, both H3BERTa and AntiBERTa2 embeddings yielded strongly bimodal distributions along the linear discriminant axis, with minimal overlap between classes. This consistent separation indicates that, despite differences in input scope and training objectives, both models encode sequence features that distinguish naive from memory B cell repertoires.

We next extended this analysis to antigen-binding discrimination using antibody sequences from immunized alpacas targeting the severe acute respiratory syndrome coronavirus 2 (SARS-CoV-2) spike protein, human interleukin-6 (hIL-6), and human tumor necrosis factor alpha (hTNF-α) (Figure 3). LDA of binding versus non-binding sequences revealed clear class separation for both H3BERTa and AntiBERTa2 across all antigens examined, with comparable degrees of separability. These results indicate that CDR-H3-centric embeddings produced by H3BERTa retain sequence signals associated with antigen binding in immunized repertoires, including those derived from a non-human species.

To determine whether these observations generalize beyond AntiBERTa2, we performed parallel analyses using two other full-length antibody language models: HeavyBERTa[27] and AbRoBERTa.[28] Across models, we observed the same overall pattern, where a substantial fraction of sequence-to-sequence variability was associated with V-gene usage and framework residues (Figure S4). These consistent trends indicate that the observed differences arise from input scope rather than model architecture.

Finally, we computed the pseudo-perplexity (PPL) of both models on a healthy donor BCR repertoire,[29] which approximates the relative plausibility of a sequence under the model. PPL reflects how well a sequence conforms to the learned statistical patterns of the model's training data and thus serves as a proxy for model-relative repertoire typicality. Lower PPL values indicate that the model considers a sequence highly plausible and statistically consistent with its training distribution, whereas higher PPL values suggest uncommon or out-of-distribution features. The PPL scores were moderately correlated between H3BERTa and AntiBERTa2 (Pearson r = 0.41, Spearman ρ = 0.60; Figure S5), indicating that despite differences in training

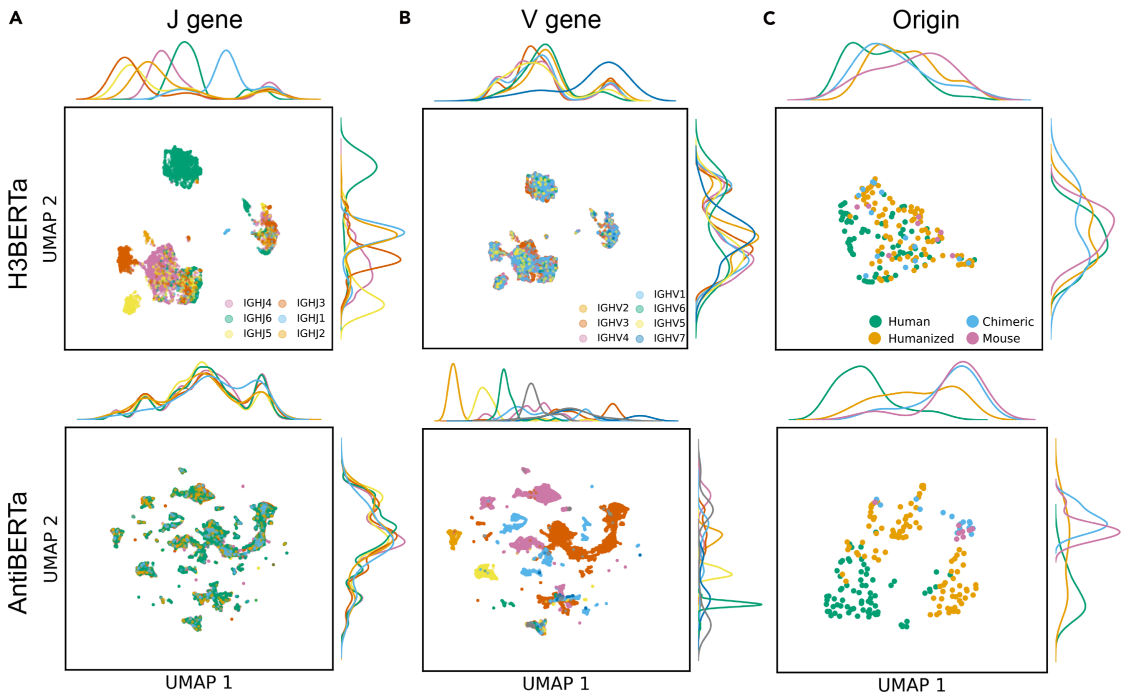

Figure 2. Comparison of antibody sequence embeddings from H3BERTa and AntiBERTa2 models

(A and B) Sequence embeddings generated by H3BERTa (top row) and AntiBERTa2 (bottom row), visualized using UMAP. Marginal distributions along each axis are shown as kernel density ridges. Each point represents a unique antibody heavy-chain sequence. UMAP of embeddings, colored by (A) IGHJ gene segment and (B) IGHV gene segment.

(C) UMAP of embeddings from therapeutic antibodies, colored by annotated species origin: human (green), humanized (orange), chimeric (light blue), and mouse (pink).

input, both models capture partially overlapping aspects of sequence composition.

Characterizing bnAb repertoires using H3BERTa

Having established that H3BERTa can organize antibody repertoires in an unsupervised manner based on intrinsic sequence features, we next investigated whether it could also detect functional differences between biologically distinct repertoires. HIV bnAb repertoires were selected as a test case because they arise from chronic antigen exposure and prolonged affinity maturation, processes expected to imprint distinct sequence characteristics on the CDR-H3 region.[30,31] If H3BERTa's embeddings capture these subtle immunogenetic signals, then they should allow discriminating between bnAb and healthy donor repertoires.

CDR-H3 sequences were analyzed from a published dataset,[29] which includes 4.4 million sequences from 42 healthy donors and 4.2 million sequences from 46 bnAb donors. For an initial overview, we selected four representative donors from each group. In the H3BERTa embedding space, donors showed a comparable overall organization, with UMAPs consistently revealing three distinct clusters corresponding to IGHJ6, IGHJ4, and IGHJ3 (Figure S6), consistent with the germline-driven structure observed in earlier analyses. No clear clustering pattern was observed when stratifying by V-gene usage (Figure S7). However, no clear group-level separation was observed in the CDR-H3-based embedding space. This indicated that potential bnAb-associated repertoire differences either are not readily visible in unsupervised embedding space or are not fully captured when modeling CDR-H3 in isolation, potentially requiring either more sensitive quantitative analyses or models that incorporate additional sequence context.

To move beyond qualitative visualization and establish a simple baseline for repertoire-level differences, we examined the distribution of CDR-H3 loop lengths. The overall length distributions were largely similar between healthy and bnAb donors (Figures S8A and S8B), with a small shift in the mean length (approximately 15.5 amino acids in healthy donors versus 15.0 in bnAb donors; Figure S8C). To quantify inter-repertoire differences, we computed pairwise Wasserstein distances between the length distributions of each repertoire. The Wasserstein distance captures differences in both distributional shape and central tendency. Using these distances for hierarchical clustering, differences in length distributions allowed a partial separation between healthy and bnAb repertoires (Figures S8D–S8G), indicating cohort-level variation in the distribution of CDR-H3 loop lengths.

To assess whether H3BERTa captures additional, higher-order sequence features, we applied the same Wasserstein-based clustering to the distributions of PPL values computed for all sequences within each repertoire. The repertoire-level distribution of PPL would allow capturing shifts in sequence composition and higher-order patterns. Hierarchical clustering based on repertoire PPL distributions showed a clearer separation between healthy and bnAb cohorts (Figure 4A). Consistently, the mean Wasserstein distance between healthy and bnAb

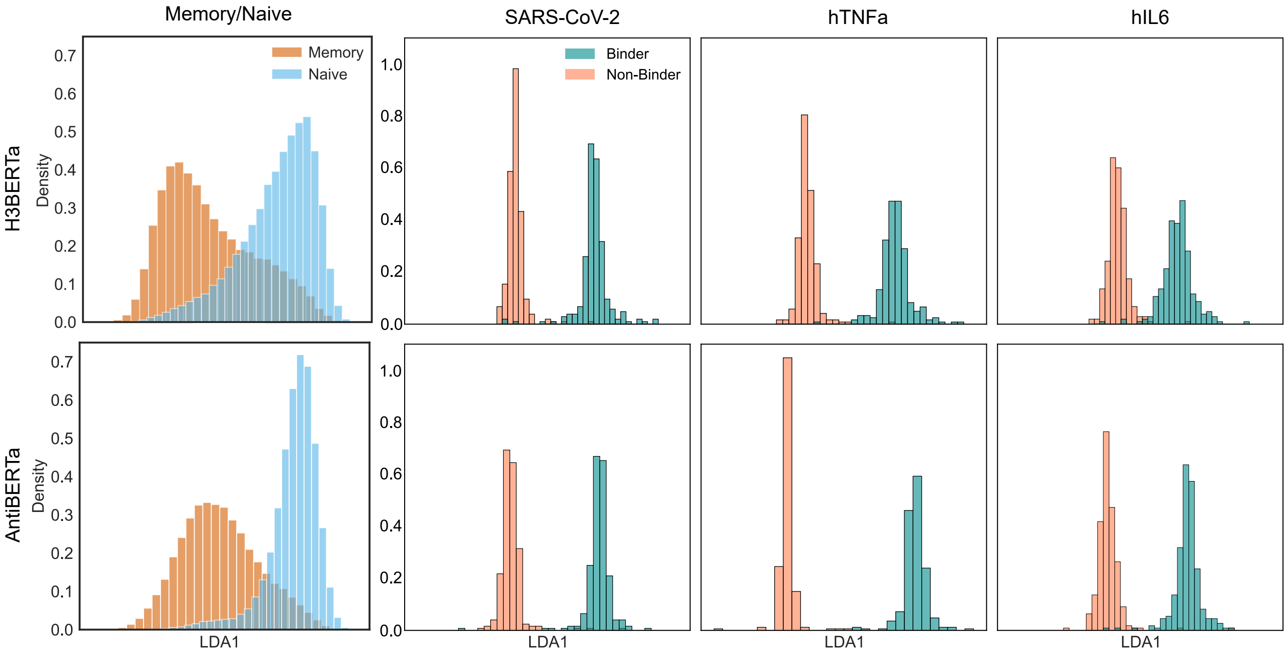

Figure 3. Linear discriminant analysis of antibody sequence embeddings generated by H3BERTa and AntiBERTa2

Sequence embeddings generated by H3BERTa (top row) and AntiBERTa2 (bottom row) were projected onto the linear discriminant axis using linear discriminant analysis (LDA). Density bar plots show the distribution of embeddings for naive and memory B cell sequences (red and blue, respectively) and for binding and non-binding antibody sequences (orange and teal, respectively). Binding classification was performed for antibodies targeting the severe acute respiratory syndrome coronavirus 2 (SARS-CoV-2) spike protein, human tumor necrosis factor alpha (hTNFα), and human interleukin-6 (hIL-6).

repertoires exceeded the mean within-group distances (Figure S9A), indicating that repertoire-wide PPL reflects systematic differences between donor cohorts.

Within the healthy group, five distinct clusters were observed, each with low internal variability, indicating a higher degree of repertoire similarity within clusters (Figures 4A and S9B). The largest cluster included approximately half of the healthy donors. Two additional clusters, each comprising approximately one-quarter of the healthy individuals, showed modest divergence from the main group. Finally, two small clusters, each containing only a few donors, stood out, with PPL profiles that were distinctly different from the rest of the healthy cohort and from all bnAb donors. These outlier groups may reflect unusual immune histories or idiosyncratic repertoire development.

In the bnAb cohort, most donors grouped into two primary clusters with internally consistent PPL profiles. The intra-cluster distances were similar to those in the healthy group, whereas the average distance between the two bnAb clusters was higher, thus contributing to an overall increase in within-group variability (Figure S9). Interestingly, three healthy donors co-clustered with bnAb repertoires (Figure 4A), indicating a partial convergence or overlap in sequence plausibility profiles. These cases may reflect repertoire features that are atypical relative to the model's learned healthy distribution, potentially resembling sequence patterns reported in bnAb-associated repertoires.

Differences in the distributional tails of PPL

Building on the observed clustering of repertoire-level PPL profiles, we next investigated whether these differences could be localized to specific regions of the distribution. Visual inspection of the merged PPL distributions revealed broad overlap between healthy and bnAb repertoires, particularly in the central range (Figure S10). The 5th–95th percentile intervals were nearly identical between groups (healthy: [2.22, 16.61]; bnAb: [2.23, 16.41]), suggesting that any group-specific divergence may lie in the distributional tails. To test this hypothesis, we focused on the lower and upper tails of the PPL distributions, defined as sequences falling outside the central 90% range. First, the non-parametric Kolmogorov-Smirnov (KS) test was applied separately to the upper and lower tails. Both comparisons revealed statistically significant deviations from the null hypothesis (upper tail: stat = 0.032, $p < 0.001$; lower tail: stat = 0.057, $p < 0.001$), indicating that the shape of the distribution in the extremes differs between groups, with the effect more pronounced in the lower tail. Importantly, directional testing using the greater and less alternatives showed that, in the upper tail, healthy repertoires tend to exhibit higher PPL values than diseased repertoires. To complement this, the Mann-Whitney U test was used to assess whether the tails differed in location. This test also revealed significant group-level differences (upper tail: $U = 2.41 \times 10^{10}$, $p < 0.001$; lower tail: $U = 2.18 \times 10^{10}$, $p < 0.001$), confirming that the group differences are concentrated in the tails rather than the center of the distribution. These results demonstrate that the observed differences are concentrated in the tails of the distribution and are directionally biased, with healthy repertoires showing higher PPL in the upper tail, while the lower tail differences reflect an opposite or more complex pattern.

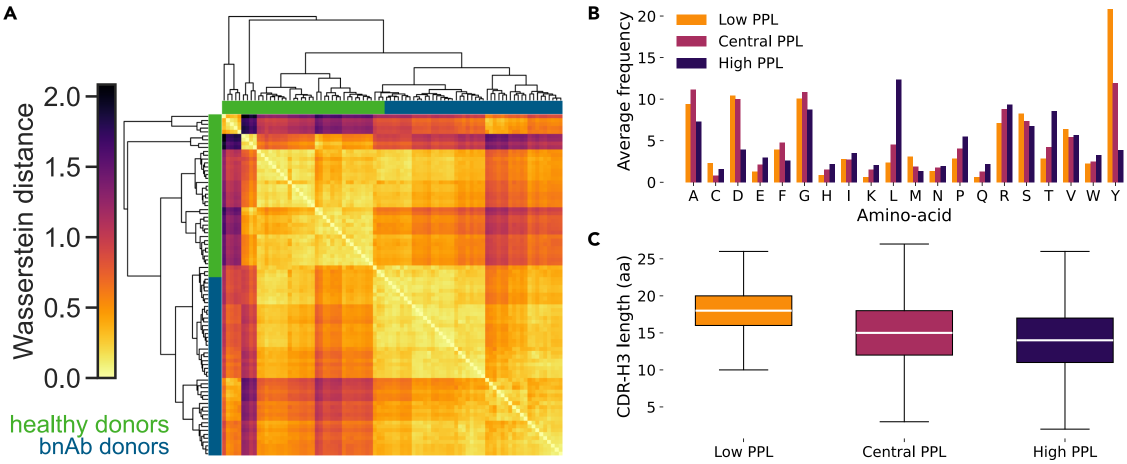

Figure 4. Pseudo-perplexity distributions of antibody repertoires quantified with H3BERTa

(A) Hierarchical clustering of donor repertoires based on pairwise Wasserstein distances between their H3BERTa pseudo-perplexity (PPL) distributions. Columns and rows correspond to individual repertoires; color intensity reflects the distance scale shown at left (yellow, similar; purple/black, dissimilar). Side annotation: green, healthy donors; blue, donors who elicited broadly neutralizing antibodies.

(B and C) Sequence features of CDR-H3s partitioned into low-, central-, and high-PPL thirds (orange, magenta, and dark violet, respectively).

(B) Mean amino acid frequency in each bin.

(C) Boxplots of CDR-H3 length for low-, central-, and high-PPL sequences. The center line denotes the median, the box represents the interquartile range, and whiskers extend to the 5th and 95th percentiles.

Biochemical characterization of perplexity-defined CDR-H3 regions

To gain biochemical insights into the sequence properties associated with the perplexity groups, we analyzed the amino acid composition and CDR-H3 loop length in the low-, central-, and high-PPL groups (lowest 5%, middle 90%, and highest 5% of perplexity values, respectively). Amino acid composition varied systematically across these PPL groups (Figures 4B and S11). Loops categorized as low PPL were enriched in tyrosine (Y), an aromatic residue frequently involved in antigen binding. In contrast, high-PPL loops showed a marked increase in the hydrophobic amino acids leucine (L) and threonine (T), along with more moderate enrichments in the conformationally restricted proline (P). These distinct changes in composition indicate that perplexity values reflect underlying biochemical profiles of CDR-H3 sequences.

A clear trend in CDR-H3 loop length was also observed across the perplexity-defined groups (Figures 4C and S12). Low-PPL sequences were typically the longest (median = 17–18 residues), followed by central-PPL (median = 15 residues) and high-PPL sequences, which were the shortest (median = 14 residues). We observed a significant association between PPL grouping and CDR-H3 length. A Kruskal-Wallis test showed a highly significant difference across groups ($H(2) = 2.9 \times 10^5$, $p < 10^{-6}$). Post hoc Dunn tests with Holm correction confirmed that all pairwise comparisons remained significant (all adjusted $p < 10^{-6}$). Notably, H3BERTa consistently assigned central or high PPL scores to rare ultra-long CDR-H3 sequences. For example, 322 out of 8,649,880 total sequences exceeded 50 amino acids in length, with the longest reaching 77 residues, confirming their atypicality from the model's learned distribution.

To evaluate whether model-derived perplexity correlates with broader biochemical properties, we analyzed net charge at physiological pH (7.0) and hydrophobicity using the GRAVY (grand average of hydropathy) score for sequences within the low-, central-, and high-PPL bins (Figure S13; Table S1). Net charge differed significantly across all groups. Low-PPL sequences were significantly less charged than central-PPL sequences, whereas high-PPL sequences carried more charge than central ones. Although modest, the hydrophobicity differences were also statistically significant in all pairwise comparisons.

bnAb classifier

Building on our repertoire-level analyses, we next investigated whether H3BERTa could also capture functional differences at the single-sequence level. Specifically, we evaluated whether its learned representations could support the classification of individual CDR-H3 loops based on their HIV-1 neutralization capacity. Therefore, a labeled dataset of 390 experimentally validated CDR-H3 sequences, with an equal number of broadly neutralizing and non-neutralizing sequences, was constructed. The bnAb sequences were sourced from the Los Alamos National Laboratory's Compile, Analyze and Tally nAb Panels (CATNAP) database,[32] a curated repository of HIV antibody neutralization data. The non-neutralizing antibody (nnAb) class combined CDR-H3s from individuals infected with HIV without broadly neutralizing activity and carefully selected "hard negatives" from healthy donors in the OAS database. These hard negatives, which share high sequence similarity with known bnAbs, were included to create a more challenging and realistic classification task. Despite this, the bnAb group exhibited a broader and right-shifted CDR-H3 length distribution relative to nnAbs, consistent with the known enrichment of long loops in bnAbs (Figure S14).

Support vector machine

We trained a linear-kernel support vector machine (SVM) using H3BERTa-derived embeddings as input features. This simple

CellPress
OPEN ACCESS

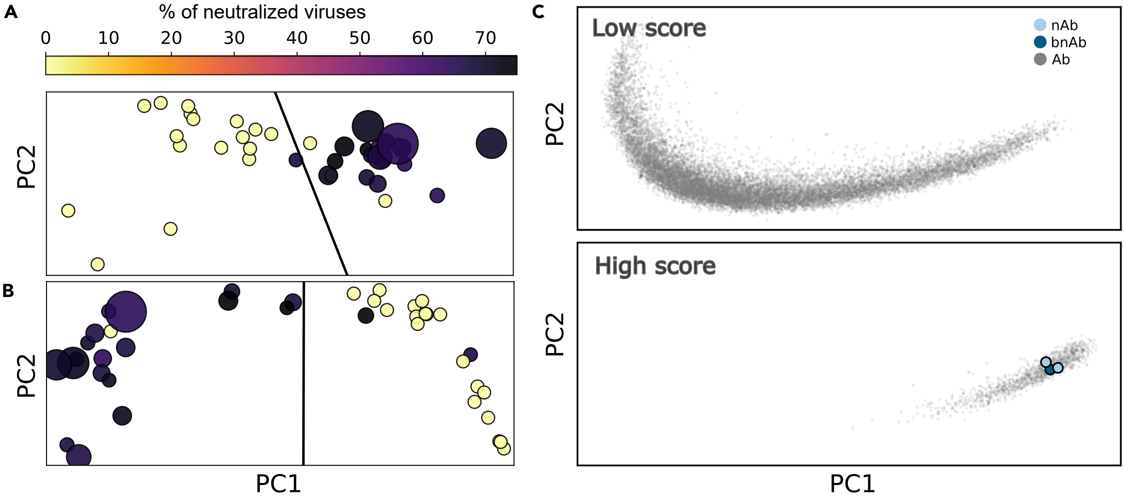

Figure 5. Antibody neutralization performance visualized through embedding-based decision boundaries and repertoire projection

(A and B) Decision boundaries (black line) of the support vector machine (SVM) classifier trained on embeddings from (A) H3BERTa and (B) GAN-H3BERTa. Each dot represents an antibody from the test set. Dot size reflects the size of the viral panel on which the antibody was tested, while the color indicates the neutralization breadth, i.e., the number of viruses neutralized by the antibody divided by the total number of viruses in the assay.

(C) PCA projection of GAN-BERT embeddings from an antibody repertoire of a donor with neutralizing serum.[33] Each dot represents an antibody from the donor's repertoire. The top image shows antibodies assigned low neutralization scores by the GAN-H3BERTa model, while the bottom image shows those assigned high scores. Blue and light blue dots correspond to antibodies predicted and experimentally characterized as bnAbs and nAbs,[33] respectively, and were also correctly identified as such by the GAN-H3BERTa approach.

and interpretable model served as an initial probe to test whether the learned representations encode sufficient discriminative information for distinguishing bnAbs from nnAbs at the sequence level.

Model performance was first evaluated on a validation set, achieving an overall accuracy of 92% on the bnAbs class and a macro-averaged F1-score of 0.92 (Table S2), indicating balanced performance across classes.

On an independent test set, performance remained consistent, with an overall accuracy of 93% and a macro F1-score of 0.92 (Table S2). This corresponded to correct classifications of 95.0% of nnAbs and 90.0% of bnAbs, with a slightly higher false negative rate observed for bnAbs (10.0%) than for nnAbs (5.0%). Visualization of the decision boundary in PCA-reduced H3BERTa embedding space confirmed that most sequences were linearly separable, with misclassifications occurring primarily near the margin (Figure 5A).

GAN-H3BERTa

To explore whether a semi-supervised deep learning approach could provide an alternative way to control the false positive rate observed with the SVM, we adopted a semi-supervised framework suited to settings with limited labeled data. Specifically, we employed generative adversarial network (GAN)-BERT,[34] which integrates a pretrained language model with adversarial fine-tuning to exploit both labeled and unlabeled data during training. Unlike conventional supervised models, GAN-BERT is designed to operate in settings with a small number of labeled examples by leveraging distributional information from a larger pool of unlabeled sequences. In our adaptation, the original BERT encoder was replaced with H3BERTa, and the model was fine-tuned using labeled CDR-H3 sequences and unlabeled sequences from the repertoires of individuals infected with HIV (Figure 1B). To construct the unlabeled set, we sampled CDR-H3 loops from antibody repertoires of HIV-positive donors with varying degrees of neutralization breadth. To enrich for functionally mature antibodies, only heavy-chain sequences with <85% V-gene identity were retained, consistent with the high levels of somatic hypermutation typically observed in HIV-related immune responses.

The GAN-H3BERTa model achieved 83.3% accuracy on the validation set (Matthews correlation coefficient [MCC]: 0.67, area under the curve [AUC]: 0.83), showing stable generalization and a reasonable balance between sensitivity and precision across classes (Table S3). On the independent test set, accuracy rose to 85.0% (MCC: 0.71, AUC: 0.85), with bnAbs detected at high sensitivity (recall: 0.95) while maintaining acceptable precision (0.79) (Table S3).

To assess robustness with respect to stochastic initialization, the model was independently trained three times with identical hyperparameters but different random seeds. While validation metrics exhibited some variability (Table S4), test performance remained comparatively stable across seeds. Overall, these results indicate limited sensitivity of the observed performance to seed-induced stochasticity.

To evaluate how adversarial fine-tuning reshaped the embedding space, we trained a linear SVM on the CDR-H3 embeddings extracted from the fine-tuned H3BERTa encoder within GAN-H3BERTa. This allowed for comparison of the separability of antibody classes in the fine-tuned space to that of the original, static H3BERTa embeddings. On the validation set, the SVM trained on GAN-BERT embeddings achieved an accuracy of 92% and a macro-averaged F1-score of 0.92, indicating strong and balanced performance across classes. Misclassifications were limited, with less than 6% of bnAbs labeled as nnAbs, and 11% false positives

in the nnAb group. These results were consistent on the test set, where accuracy reached 93% and the macro F1-score remained at 0.92. Class-level precision and recall also remained balanced, with slightly improved recall for nnAbs and precision for bnAbs. Overall, both the validation and test sets indicate that adversarial fine-tuning reshapes the embedding space in a way that supports clearer linear class separation, even when a simple classifier such as an SVM is applied. Although both SVMs showed structured decision boundaries (Figures 5A and 5B), the latent space learned by GAN-H3BERTa appeared to be more structured and further from the decision margin, suggesting that semi-supervised training contributed to a more structured and discriminative feature space.

Finally, to assess model performance under more realistic conditions, we applied both the SVM and GAN-H3BERTa classifiers to the complete B cell repertoire from a donor infected with HIV-1 known to produce bnAbs,[33] which includes experimentally characterized antibodies. This analysis allows evaluation of whether the models can correctly identify known bnAbs while estimating the overall specificity in a full repertoire. Overall, the SVM predicted 31.1% of sequences as bnAbs, while the GAN-H3BERTa approach reduced this slightly to 28.0% of sequences, reflecting a modest shift in predicted class proportions. Importantly, both models correctly classified the experimentally validated antibodies, one bnAb and two neutralizers, as bnAbs. In latent space, these sequences clustered within high-confidence bnAb regions (Figure 5C), and HIV-1-specific sequences more broadly localized to the same region, suggesting that H3BERTa captures relevant antigen-associated features even in complex, unfiltered repertoires. Taken together, these results indicate that the primary contribution of GAN-H3BERTa in this setting lies in exploratory semi-supervised representation learning and embedding refinement rather than in consistent performance gains over simpler linear classifiers.

DISCUSSION

The identification of antigen-specific antibodies within the vast and heterogeneous BCR repertoire remains a major challenge in immunology, vaccinology, and therapeutic antibody discovery. Although next-generation sequencing has enabled unprecedented access to antibody repertoires, extracting functionally relevant candidates from this immense sequence space remains difficult, particularly in the absence of antigen-specific labeling. Recent computational approaches, including machine-learning-based models, have sought to identify patterns in antibody sequences[1–4]; most rely on full-length variable-region sequences. Self-supervised pretraining of such models can capture broad sequence representations. However, the larger parameter space and broader sequence coverage increase the amount of labeled data and computational resources required for downstream fine-tuning, as the model must learn to focus on task-relevant features within a much richer representation space.

We hypothesized that focusing exclusively on the hypervariable CDR-H3 region could retain much of the relevant immunological signal while reducing the complexity of the representation space. This would enable effective downstream analysis even in low-label or fragmentary data settings. We developed H3BERTa, a transformer-based language model trained on over 18 million CDR-H3 sequences, to test whether this hypervariable region alone could capture meaningful immunological signals in antibody repertoires. Beyond computational efficiency, a CDR-H3-focused language model offers several practical advantages. Since CDR-H3 is the most variable and least species-specific region of the antibody, H3BERTa can be applied more readily across datasets from different organisms than full-length models whose representations depend on species-specific framework signals. Focusing exclusively on CDR-H3 also isolates the primary antigen-contact region, yielding representations that emphasize biophysically meaningful properties such as loop length, hydrophobicity, and charge. This makes the learned features interpretable and directly tied to antigen-binding potential. In addition, the reduced sequence length makes H3BERTa scalable to full-repertoire analyses, where millions of sequences can be processed efficiently, an important practical requirement for immunological applications. Finally, CDR-H3-centric models are complementary to full-length antibody language models, capturing loop-level diversity rather than germline- or framework-driven signals, and thus provide a distinct and useful perspective on repertoire structure.

Our findings show that despite the reduced input scope, H3BERTa successfully recapitulated many biologically coherent repertoire-level features. The most prominent structure in H3BERTa's latent space was clustering by J-gene usage, reflecting the contribution of IGHJ segments to the C-terminal residues of CDR-H3 and, more broadly, the influence of J-gene choice on loop length and amino acid composition. These sequence-level effects shape the physicochemical properties of the CDR-H3 loop that are relevant for antigen recognition.[35]

To assess whether H3BERTa embeddings capture functional signals beyond sequence composition, we tested whether the B cell maturation state could be inferred from the latent representations using a simple LDA. Although maturation is associated with the accumulation of somatic hypermutations, CDR-H3 sequences are short and highly diverse, making the extraction of relevant information challenging. The ability to discriminate maturation states from these representations indicates that H3BERTa encodes functionally meaningful features within the hypervariable region, supporting its applicability for repertoire-level analyses. Although some separation between populations may arise from J-gene usage and other maturation-linked sequence features, the fact that these distinctions can be captured using only CDR-H3 sequences underscores the model's capacity to capture functional, maturation-linked repertoire information.

H3BERTa also enables fine-grained comparison of PPL distributions, providing a complementary view of sequence diversity and repertoire-level variation within antibody repertoires. Although healthy and HIV-1 repertoires largely overlap in PPL, statistical tests revealed consistent differences, especially at distribution extremes. It is known that next-generation sequencing captures only a small fraction of the total B cell repertoire.[36] Nonetheless, since these distributional differences are observed systematically across samples, they are unlikely to arise from random sampling effects and may instead reflect underlying biological variation. Further work will be needed to determine the immunological processes contributing to these patterns.

Low-PPL sequences tend to have longer CDR-H3 loops enriched in tyrosine residues, features often associated with greater structural complexity and observed in the context of affinity maturation.[37] In contrast, high-PPL sequences more frequently display shorter, leucine-rich CDR-H3s, a pattern consistent with sequence features reported in less mature or more conformationally permissive binding configurations.[38] The presence of these distributional differences between healthy and HIV-1 repertoires is consistent with differences in B cell selection pressures and repertoire shaping under chronic antigen exposure.[39–42]

It should be noted that these divergences cannot be explained solely by CDR-H3 length or amino acid composition, indicating that H3BERTa captures additional sequence-level features beyond simple compositional biases. We also observed that sequences with higher net positive charge tend to exhibit greater PPL, implying that electrostatic properties influence model uncertainty. This observation is qualitatively consistent with reports that affinity maturation and antigen engagement involve changes in surface charge and loop dynamics.[43] Together, these results suggest that H3BERTa's PPL distributions capture statistically meaningful biophysical and evolutionary signatures present in antibody repertoires, offering a sensitive statistical framework for comparing antibody repertoires even when only limited sequence data are available.

The effectiveness of H3BERTa embeddings in downstream classification tasks demonstrates that the model captures discriminative, task-relevant information within the CDR-H3 region. Even a simple linear SVM achieved strong separation between neutralizing antibodies (nAbs) and nnAbs, highlighting the biological richness of the learned representations.

When the SVM classifier was applied to full B cell repertoires, its limitations became evident. Although it performed well on balanced validation sets, it generated many false positives in realistic repertoires, where bnAbs constitute only a small fraction of all sequences.[8] This outcome illustrates a common challenge in repertoire-level prediction: extreme class imbalance and biological heterogeneity can cause apparent model performance to degrade when moving from curated datasets to natural repertoires. To mitigate this, we explored a semi-supervised GAN-BERT framework that fine-tunes H3BERTa's embedding space using limited labeled data. This approach improved the separability of bnAb sequences and modestly reduced false positives, demonstrating the potential of semi-supervised strategies for antibody classification under data-scarce conditions. While additional data and further methodological development will be required to achieve stronger discrimination, these results suggest that integrating generative and discriminative learning could be a fruitful direction for future repertoire modeling efforts.

In conclusion, these results demonstrate that H3BERTa captures not only germline- and structure-driven aspects of antibody repertoires but also subtler evolutionary and biophysical signals associated with affinity maturation and immune history. By distilling immunologically meaningful representations from the hypervariable CDR-H3 region, H3BERTa provides a compact yet informative framework for analyzing repertoire dynamics across healthy and diseased donors. Such representations hold promise for advancing antibody discovery, vaccine design, and longitudinal immune monitoring. Future work will focus on evaluating H3BERTa across broader datasets and disease contexts, assessing its capacity to predict antigen specificity and neutralization breadth directly from sequence, and refining the GAN-BERT framework to improve the detection of rare, therapeutically relevant antibodies in large-scale repertoires.

Limitations of the study

Although H3BERTa provides a compact and informative representation of antibody repertoires, several limitations should be considered. First, by focusing exclusively on the CDR-H3 region, the model does not explicitly capture contributions from other CDRs or framework residues that can influence antigen binding and structural stability. Second, although the model performs well at discriminating functional states and sequence-level properties, its predictive performance for rare or low-frequency antibodies in full repertoires is constrained by class imbalance and the limited availability of labeled data. Third, H3BERTa has been trained and evaluated primarily on humans; its generalizability to other species or heavily engineered antibodies remains to be fully tested. Finally, while PPL and embedding-based analyses provide valuable insights into sequence- and repertoire-level features, they are indirect measures and will benefit from complementary experimental validation. Addressing these points will help further expand H3BERTa's applicability in antibody discovery, immune monitoring, and cross-species repertoire analyses.

METHODS

H3BERTa

H3BERTa is a transformer-based language model designed specifically for CDR-H3 loop sequences of BCRs. The model was trained on sequences sourced from the OAS database.[20]

Dataset

The H3BERTa training dataset was constructed by extracting the CDR-H3 sequences from the OAS database,[20] specifically selecting entries from healthy human donors with no documented history of vaccination or disease, and including all available B cell sources (Figure 1A). IgG and IgA isotype sequences were extracted, and CDR-H3 loops were identified using IMGT annotations. To reduce redundancy, the CDR-H3 sequences were clustered at 70% identity using MMseqs2,[44] retaining only the cluster centroids. CDR-H3s containing more than two ambiguous amino acids (e.g., "X") or with CDR-H3 loops shorter than three residues were excluded, resulting in a final dataset of approximately 18 million sequences. The remaining centroids were further clustered at 30% identity to create three distinct splits: training (80%), validation (10%), and testing (10%). To ensure data consistency, all sequences within a 30% identity cluster were assigned to the same split, with larger clusters preferentially allocated to the training set.

Model

We used the RoBERTa architecture[19] as the base model and conducted an extensive architecture search to explore variations in model depth and complexity. The search involved adjusting the hidden size (from 512 to 768), the intermediate size (from 2,048 to 3,072), the number of attention heads (from 4 to 12), and the number of attention layers (from 4 to 12). This exploration

resulted in a range of model sizes, with the number of trainable parameters varying from 16 million to 86 million. The final model configuration features a hidden size of 768, an intermediate size of 3,072, 12 attention heads, and 12 attention layers, totaling 86 million parameters. Sequences were tokenized at the amino acid level, with a maximum sequence length fixed at 100 residues.

Training

The batch size was set to 1,024, and we tested learning rates of 1×10^{-3}, 5×10^{-5}, and 1×10^{-6}, with 5×10^{-5} emerging as optimal. Training was performed using the AdamW optimizer with a weight decay of 0.1 and took approximately 1 month on a single A100 80G NVIDIA GPU to reach convergence at epoch 113.

Classifier

bnAb dataset

We compiled our labeled dataset using information from multiple studies available in the Los Alamos National Laboratory's HIV Database (CATNAP).[32] Our selection was restricted to human-derived data.

We categorized antibodies into two groups.

- bnAbs: antibodies with an average neutralization detection rate exceeding 25%.
- nnAbs: antibodies with no detectable neutralization activity.

We retrieved available nucleotide sequences and processed them using the Python package PyIR,[45] isolating the CDR-H3 based on IMGT annotations. To mitigate initial class imbalance and introduce challenging negative examples, we enriched the nnAb category by including CDR-H3 sequences from healthy individuals from OAS that shared over 70% sequence identity with bnAbs retrieved using blastp with the following default parameters: -evalue 2e-5 -outfmt 6 -word_size 2 -threshold 11 -matrix PAM30 -window_size 40 -comp_based_stats 0. This resulted in a perfectly balanced dataset comprising 195 sequences per class. To prevent information leakage, we used MMseqs2, specifically the Linclust algorithm with default parameters, to cluster sequences within each antibody category prior to splitting. The resulting clusters were then used to partition the dataset into training (80%), validation (10%), and test (10%) subsets using a standard split strategy. All sequences within a given cluster were assigned to the same dataset split, with larger clusters preferentially allocated to the training set to ensure sufficient representation, while preserving the independence of the validation and test sets. Additionally, each split was randomly shuffled to eliminate potential ordering effects before training and evaluation.

SVM

We employed the pretrained H3BERTa model, which was trained on healthy antibody repertoires, as a fixed embedding extractor. Each input CDR-H3 sequence was tokenized at the single-residue level and fed through the model. To obtain a fixed-length embedding for each sequence, we applied mean pooling over the final hidden states, averaging only over positions corresponding to valid (non-padded) tokens as indicated by the attention mask. The resulting embeddings, representing contextualized sequence-level features, were subsequently used to train a linear SVM classifier. The classifier was implemented using the scikit-learn library, trained on the designated training set, and evaluated on a held-out test set to assess generalization performance. For comparison, we repeated the entire procedure using the pretrained AntiBERTa2 model as a fixed embedding extractor.

GAN-BERT

For the detection of bnAbs, we employed the GAN-BERT approach,[34] an adversarial classifier that combines a pretrained language model with adversarial training. This method leverages both a small labeled dataset and a larger unlabeled dataset.

Labeled dataset

For the labeled dataset, we used the annotated data to train, validate, and test the SVM classifier.

Unlabeled dataset

Antibody heavy-chain repertoire sequences were obtained from a previously published dataset,[29] comprising 139 individuals with chronic HIV-1 infection (46 with broad neutralization breadth and 50 with limited breadth) and 43 HIV-uninfected controls. Repertoires were labeled according to donor-level neutralization phenotype: bnAbs (broad neutralization), nAbs (limited neutralization), and nnAbs (HIV-negative controls/healthy donors). In this study, we employed only bnAb and nnAb phenotypes.

Sequences were filtered by V-gene germline identity, retaining only those below 85%. Unique CDR-H3 loops were extracted and used as a dataset.

Two configurations of the unlabeled dataset were constructed. In the balanced version, approximately 10,000 sequences from each of the bnAb and nAb groups were combined with ~20,000 randomly sampled sequences from the nnAb group. In the HIV-positive-only version, only bnAb and nAb repertoires were included (~10,000 sequences per group), assuming the majority of sequences in these repertoires to be non-neutralizing.

Model

We implemented a semi-supervised learning model based on the GAN-BERT framework,[34] utilizing the Hugging Face transformers library. Our model employs H3BERTa as the language model backbone and integrates a GAN comprising a generator (G) and a discriminator (D).

The G is a multilayer feedforward neural network. It takes as input a random vector of size 1,000, sampled from a normal distribution, and produces "fake" embedding vectors of dimension 768, matching the dimensionality of the real H3BERTa embeddings. The G architecture consists of 1 hidden fully connected layer with 768 units, followed by a LeakyReLU activation function (with negative slope 0.01) and a dropout layer with a rate of 0.1.

The D is a multilayer feedforward neural network that takes as input either a real embedding from H3BERTa (corresponding to a CDR-H3 sequence) or a fake embedding generated by G. Its output is a three-way classification, distinguishing between the nnAb and bnAb categories, as well as a third category representing the fake embeddings generated by G. The D architecture consists of 2 fully connected layers with 768 units each, each followed by a LeakyReLU activation and dropout (rate 0.1).

The H3BERTa model was fine-tuned during training. The real embeddings used as input to the D were obtained by passing the CDR-H3 sequences through the H3BERTa model. Both G and D were optimized using the Adam optimizer with a learning rate of 5×10^{-5} and epsilon = 1×10^{-8}. The model was trained

across 1 GPU without a learning rate scheduler with a warmup proportion of 0.1.

Training

We conducted a hyperparameter search to optimize our model's performance. The following hyperparameters were explored.

- Learning rates: 5×10^{-5} and 5×10^{-6}.
- Hidden layers in both the G and D: 1, 2, and 3 layers.
- G input dimensionality (noise size): 10, 50, 100, 500, and 1,000.
- Dropout rate: 0.0, 0.1, 0.2, and 0.3 applied at the output layer of the D.
- Batch size: 16, 32, 64, 128, and 256.

The final model was trained on a shuffled version of the balanced, unlabeled dataset described for the unsupervised objective and on the labeled dataset introduced for the supervised objective. Sequences were tokenized at the amino acid level, with a maximum sequence length of 100 residues.

Training was conducted using adversarial objectives for both G and D, combined with a supervised cross-entropy loss applied to labeled data. The D loss was defined as the sum of the supervised and unsupervised terms, while the G loss included both adversarial and feature-matching components.

The optimal configuration, selected based on the lowest validation loss, comprised a batch size of 16, learning rates of 5×10^{-5} for both the G and the D, one hidden layer in the G, two hidden layers in the D, a noise vector size of 1,000, and a dropout rate of 0.1. Model training was performed on a single NVIDIA A100 80GB GPU.

Evaluation data

For all analyses involving stochastic components, such as UMAP dimensionality reduction, a fixed random seed of 42 was applied to ensure full reproducibility of the results.

Single repertoire analysis

From the same unlabeled dataset used to train the GAN-BERT classifier, we selected one repertoire from each phenotype: one from the healthy group (ID: 700011206, number of not unique sequences: 113,450) and one from the bnAb group (ID: 702010293, number of not unique sequences: 115,719).

Naive/memory B cells and CDR-L3/V-gene features

The dataset used to extract embeddings of naive and memory B cells (heavy chain) and of the CDR-L3 region, V-gene family, and locus usage (light chain) consists of paired heavy- and light-chain antibody sequences extracted from the OAS database. Human entries were first downloaded, and duplicates were removed based on the heavy-chain CDR-H3 sequence. Naive versus memory labels are therefore assigned according to the heavy-chain annotation. For each retained heavy chain, the corresponding paired light-chain sequence was kept, enabling downstream embedding analyses focused on the light chain (including CDR-L3, V-gene family, and locus). A balanced dataset was then constructed by randomly selecting 50,000 unique heavy chains from naive B cells and 50,000 from memory B cells. A fixed random seed was applied to guarantee reproducibility.

Binding/non-binding alpaca antibodies

The datasets used for extracting embeddings of binding antibodies were obtained from Tsuruta et al.[46,47] and accessed via the Cognanous dataset repository (https://cognanous.com/datasets). The datasets were downloaded through the Hugging Face platform.

Antibody therapeutics

All approved phase 1–3 antibody therapeutics were retrieved from Thera-SAbDab.[26] The origin of each therapeutic was determined based on its source infix and mapped to one of the following categories: human ("u"), humanized ("zu"), chimeric/humanized ("xizu"), chimeric ("xi"), or mouse ("o"). For the purposes of this analysis, therapeutics categorized as chimeric/humanized were excluded. A final dataset of 199 antibody therapeutics was used.

Immune repertoire analysis

We again employed the same collections of repertoires from the single repertoire analysis (unlabeled dataset), focusing specifically on uninfected individuals (healthy donors) and individuals infected with HIV-1 with confirmed bnAb responses. The dataset consisted of CDR-H3 immune repertoire sequences from 42 healthy and 46 HIV-1-infected donors. Unlike the classification task, no filtering or preprocessing steps were applied to the sequences prior to analysis. PPL scores for all CDR-H3 sequences were computed using our H3BERTa model, and the resulting scores were subsequently analyzed to assess repertoire-level differences between the two groups.

Quantification and statistical analysis

Embedding generation

For each CDR-H3 sequence, fixed-length embeddings were extracted from the final hidden layer of H3BERTa by mean pooling across the sequence dimension. These embeddings were used as input features for all downstream analyses, including dimensionality reduction, repertoire-level comparisons, and classification tasks.

Dimensionality reduction and visualization

Low-dimensional representations of antibody repertoires were obtained using PCA and UMAP. PCA was computed using the first two principal components to visualize the global variance structure. UMAPs were generated using cosine distance with a fixed random seed to ensure reproducibility. Embeddings were colored according to annotated features such as IGHJ gene usage, IGHV gene usage, species of origin, or experimental labels, as specified in the corresponding figure legends. To assess whether H3BERTa embeddings encode biologically meaningful categorical information, LDA was also applied to project embeddings onto a one-dimensional discriminant axis. Density distributions along the discriminant axis were visualized to assess class separation.

Comparison of H3BERTa and full-length antibody language models

For comparative analysis, we leveraged publicly available pretrained model weights, namely AntiBERTa2 (alchemab/antiberta2), Ab-RoBERTa (mogam-ai/Ab-RoBERTa), and HeavyBERTa (leaBroe/HeavyBERTa), obtained from Hugging Face.

PPL

To estimate the PPL of individual sequences using a masked language model (MLM) such as H3BERTa, we followed the token-by-token masking approach introduced by Salazar et al.[48] Given a pretrained MLM and a target sequence (CDR-H3), each non-special token in the sequence was

iteratively replaced with the model's mask token. The masked sequence was then passed through the model to compute the log probability of the true token at the masked position, conditioned on the rest of the sequence. This process was repeated for all tokens (excluding special tokens), and the final PPL was computed as the exponential of the negative average log probability:

$$\mathrm{PPL}(x) = \exp\left(-\frac{1}{N}\sum_{i=1}^{N}\log P(x_i|x_{-i})\right), \quad \text{(Equation 1)}$$

where x_i is the true token at position i and x_{-i} denotes the sequence with the ith token masked. This method provides a token-level approximation of perplexity suitable for uni/bidirectional models. The implementation was performed using PyTorch and Hugging Face transformers. Gradients were disabled during inference.

Characterizing repertoire-level distribution

To quantitatively compare antibody repertoires, distributions of CDR-H3 length and PPL were computed for each donor. Pairwise differences between repertoires were quantified using the first-order Wasserstein (earth mover's) distance. Hierarchical clustering was performed on the resulting distance matrices using average linkage, and cluster structure was visualized as heatmaps. Within- and between-group distances were compared to assess cohort-level differences. Statistical analyses were performed using Python SciPy 1.13.1.

Differences in the distributional tails of PPL

Differences in PPL distributions were assessed using the KS test to compare distributional shapes and the Mann-Whitney U test to evaluate shifts in central tendency. All tests were two-sided. Tests were applied to per-sequence distributions aggregated by repertoire. Multiple comparisons were not performed, as each test corresponds to a specific pairwise comparison. Statistical analyses were performed using Python SciPy 1.13.1.

Biochemical characterization of PPL-defined CDR-H3 regions

Amino acid composition, CDR-H3 length, net charge at physiological pH (7.0), and hydrophobicity (GRAVY score) were computed for subsets of sequences stratified by PPL bins. All analyses were performed at the per-sequence level. Group-wise comparisons involving more than two bins were assessed using Kruskal-Wallis tests, followed by post hoc Dunn tests with Holm correction for multiple comparisons. Pairwise comparisons of net charge at physiological pH (7.0) and hydrophobicity were performed using the Mann-Whitney U test. All statistical analyses were performed using Python SciPy 1.13.1.

Figures

All figures were generated directly from the analysis code without manual image manipulation beyond uniform scaling and labeling.

RESOURCE AVAILABILITY

Lead contact

Requests for further information and resources should be directed to and will be fulfilled by the lead contact, Thomas Lemmin (thomas.lemmin@unibe.ch).

Materials availability

This study did not generate new materials.

Data and code availability

- All code, pretrained models, and curated datasets used in this study are openly available to support transparency, reproducibility, and community-driven development. All data used in this study are publicly available from the OAS database (https://opig.stats.ox.ac.uk/webapps/oas/) and the CATNAP database (https://www.hiv.lanl.gov/components/sequence/HIV/neutralization/download_db.comp). The datasets used to train, validate, and test the models have been deposited on Zenodo and are accessible at https://zenodo.org/records/17505849.[49] The weights of all trained models have been deposited on Zenodo and the H3BERTa weights on Hugging Face (https://github.com/ibmm-unibe-ch/H3BERTa) and are publicly available as of the date of publication.
- Any additional information required to reanalyze the data reported in this article is available from the lead contact upon request.

ACKNOWLEDGMENTS

This work was supported by funds from the Helmut Horten Stiftung Young Researcher Grant (2022-YIG-089) and the Swiss National Science Foundation (PCEFP3 194606).

AUTHOR CONTRIBUTIONS

Conceptualization, C.R. and T.L.; methodology, C.R. and T.L.; investigation, C.R.; writing – original draft, C.R. and T.L.; writing – review & editing, C.R. and T.L.; funding acquisition, T.L.; resources, C.R. and T.L.; supervision, T.L.

DECLARATION OF INTERESTS

C.R. conducted this research while working at the University of Bern and is currently employed at the Botnar Institute of Immune Engineering.

DECLARATION OF GENERATIVE AI AND AI-ASSISTED TECHNOLOGIES IN THE WRITING PROCESS

During the preparation of this manuscript, the authors employed generative AI tools, including ChatGPT (OpenAI) and Gemini (Google), to refine the language. These tools were used solely to improve clarity, coherence, and style. All AI-assisted content was carefully reviewed and substantively edited by the authors, who accept full responsibility for the accuracy and integrity of the final submitted work.

SUPPLEMENTAL INFORMATION

Supplemental information can be found online at https://doi.org/10.1016/j.patter.2026.101561.

Received: October 21, 2025
Revised: February 6, 2026
Accepted: April 20, 2026
Published: May 20, 2026

REFERENCES

1. Ruffolo, J.A., Gray, J.J., and Sulam, J. (2021). Deciphering antibody affinity maturation with language models and weakly supervised learning. Preprint at arXiv. https://doi.org/10.48550/arXiv.2112.07782.
2. Leem, J., Mitchell, L.S., Farmery, J.H.R., Barton, J., and Galson, J.D. (2022). Deciphering the language of antibodies using self-supervised learning. Patterns *3*, 100513. https://doi.org/10.1016/j.patter.2022.100513.

CellPress
OPEN ACCESS

3. Shuai, R.W., Ruffolo, J.A., and Gray, J.J. (2023). IgLM: Infilling language modeling for antibody sequence design. Cell Syst. *14*, 979–989.e4. https://doi.org/10.1016/j.cels.2023.10.001.

4. Kenlay, H., Dreyer, F.A., Kovaltsuk, A., Miketa, D., Pires, D., and Deane, C.M. (2024). Large scale paired antibody language models. PLoS Comput. Biol. *20*, e1012646. https://doi.org/10.1371/journal.pcbi.1012646.

5. Norman, R.A., Ambrosetti, F., Bonvin, A.M.J.J., Colwell, L.J., Kelm, S., Kumar, S., and Krawczyk, K. (2020). Computational approaches to therapeutic antibody design: established methods and emerging trends. Brief. Bioinform. *21*, 1549–1567. https://doi.org/10.1093/bib/bbz095.

6. Lees, W.D., and Shepherd, A.J. (2017). Studying antibody repertoires with next-generation sequencing. In Methods in Molecular Biology vol. 1526 of Methods in Molecular Biology, K.A. Howard and M.H.M. Nielsen, eds. (Humana Press), pp. 257–270. https://doi.org/10.1007/978-1-4939-6613-4_15.

7. Franz, B., May, K.F., Jr., Dranoff, G., and Wucherpfennig, K. (2011). Ex vivo characterization and isolation of rare memory B cells with antigen tetramers. Blood *118*, 348–357. https://doi.org/10.1182/blood-2011-03-341917.

8. Mahendra, A., Haque, A., Prabakaran, P., Mackness, B.C., Fuller, T.P., Liu, X., Kathuria, S.V., Wang, Y.H., Amatya, N., Yu, X., et al. (2022). Honing-in antigen-specific cells during antibody discovery: a user-friendly process to mine a deeper repertoire. Commun. Biol. *5*, 1157. https://doi.org/10.1038/s42003-022-04129-7.

9. Chen, Y., Zhao, X., Zhou, H., Zhu, H., Jiang, S., and Wang, P. (2023). Broadly neutralizing antibodies to SARS-CoV-2 and other human coronaviruses. Nat. Rev. Immunol. *23*, 189–199. https://doi.org/10.1038/s41577-022-00784-3.

10. Chen, Y., Wang, F., Yin, L., Jiang, H., Lu, X., Bi, Y., Zhang, W., Shi, Y., Burioni, R., Tong, Z., et al. (2022). Structural basis for a human broadly neutralizing influenza A hemagglutinin stem-specific antibody including H17/18 subtypes. Nat. Commun. *13*, 7603. https://doi.org/10.1038/s41467-022-35236-y.

11. Thavarajah, J.J., Hønge, B.L., and Wejse, C.M. (2024). The use of broadly neutralizing antibodies (bNAbs) in HIV-1 treatment and prevention. Viruses *16*, 911. https://doi.org/10.3390/v16060911.

12. Landais, E., and Moore, P.L. (2018). Development of broadly neutralizing antibodies in HIV-1-infected elite neutralizers. Retrovirology *15*, 61. https://doi.org/10.1186/s12977-018-0443-0.

13. Guthmiller, J.J., Han, J., Utset, H.A., Li, L., Lan, L.Y.L., Henry, C., Stamper, C.T., McMahon, M., O'Dell, G., Fernández-Quintero, M.L., et al. (2022). Broadly neutralizing antibodies target a haemagglutinin anchor epitope. Nature *602*, 314–320. https://doi.org/10.1038/s41586-021-04356-8.

14. Tebas, P. (2025). Future of bNAbs in HIV treatment. Curr. HIV AIDS Rep. *22*, 34. https://doi.org/10.1007/s11904-025-00744-1.

15. Doria-Rose, N.A., and Landais, E. (2019). Coevolution of HIV-1 and broadly neutralizing antibodies. Curr. Opin. HIV AIDS *14*, 286–293. https://doi.org/10.1097/COH.0000000000000550.

16. Bruel, T., Guivel-Benhassine, F., Amraoui, S., Malbec, M., Richard, L., Bourdic, K., Donahue, D.A., Lorin, V., Casartelli, N., Noël, N., et al. (2016). Elimination of HIV-1-infected cells by broadly neutralizing antibodies. Nat. Commun. *7*, 10844. https://doi.org/10.1038/ncomms10844.

17. Ivanov, I.I., Schelonka, R.L., Zhuang, Y., Gartland, G.L., Zemlin, M., and Schroeder, H.W. (2005). Development of the expressed Ig CDR-H3 repertoire is marked by focusing of constraints in length, amino acid use, and charge that are first established in early B cell progenitors. J. Immunol. *174*, 7773–7780. https://doi.org/10.4049/jimmunol.174.12.7773.

18. Imkeller, K., and Wardemann, H. (2018). Assessing human B cell repertoire diversity and convergence. Immunol. Rev. *284*, 51–66. https://doi.org/10.1111/imr.12670.

19. Liu, Y., Ott, M., Goyal, N., Du, J., Joshi, M., Chen, D., Levy, O., Lewis, M., Zettlemoyer, L., and Stoyanov, V. (2019). RoBERTa: a robustly optimized BERT pretraining approach. Preprint at arXiv. https://doi.org/10.48550/arXiv.1907.11692.

20. Olsen, T.H., Boyles, F., and Deane, C.M. (2022). Observed antibody space: a diverse database of cleaned, annotated, and translated unpaired and paired antibody sequences. Protein Sci. *31*, 141–146. https://doi.org/10.1002/pro.4205.

21. Abbas, A.K., Lichtman, A.H., and Pillai, S. (2014). Cellular and Molecular Immunology, 10th ed. (Elsevier Health Sciences).

22. Bernardes, J.P., Mishra, N., Tran, F., Bahmer, T., Best, L., Blase, J.I., Bordoni, D., Franzenburg, J., Geisen, U., Josephs-Spaulding, J., et al. (2020). Longitudinal multi-omics analyses identify responses of megakaryocytes, erythroid cells, and plasmablasts as hallmarks of severe COVID-19. Immunity *53*, 1296–1314.e9. https://doi.org/10.1016/j.immuni.2020.11.017.

23. Vázquez Bernat, N., Corcoran, M., Hardt, U., Kaduk, M., Phad, G.E., Martin, M., and Karlsson Hedestam, G.B. (2019). High-quality library preparation for NGS-based immunoglobulin germline gene inference and repertoire expression analysis. Front. Immunol. *10*, 660. https://doi.org/10.3389/fimmu.2019.00660.

24. Briney, B., Inderbitzin, A., Joyce, C., and Burton, D.R. (2019). Commonality despite exceptional diversity in the baseline human antibody repertoire. Nature *566*, 393–397. https://doi.org/10.1038/s41586-019-0879-y.

25. Barton, J., Galson, J.D., and Leem, J. (2023). Enhancing antibody language models with structural information. In Proceedings of the Machine Learning for Structural Biology Workshop at NeurIPS 2023. https://doi.org/10.1101/2023.12.12.569610.

26. Raybould, M.I.J., Marks, C., Lewis, A.P., Shi, J., Bujotzek, A., Taddese, B., and Deane, C.M. (2020). Thera-SAbDab: the therapeutic structural antibody database. Nucleic Acids Res. *48*, D383–D388. https://doi.org/10.1093/nar/gkz827.

27. Brönnimann, L., Lemmin, T., and Rodella, C. (2026). Deep generative modeling captures maturation-dependent pairing patterns in human antibodies. iScience *29*, 114447. https://doi.org/10.1016/j.isci.2025.114447.

28. Huh, E., Lee, H., and Shin, H. (2025). Antibody foundational model: Ab-RoBERTa. Preprint at arXiv. https://doi.org/10.48550/arXiv.2506.13006.

29. Roskin, K.M., Jackson, K.J.L., Lee, J.Y., Hoh, R.A., Joshi, S.A., Hwang, K.K., Bonsignori, M., Pedroza-Pacheco, I., Liao, H.X., Moody, M.A., et al. (2020). Aberrant B cell repertoire selection associated with HIV neutralizing antibody breadth. Nat. Immunol. *21*, 199–209. https://doi.org/10.1038/s41590-019-0581-0.

30. Walker, L.M., and Burton, D.R. (2010). Rational antibody-based HIV-1 vaccine design: current approaches and future directions. Curr. Opin. Immunol. *22*, 358–366. https://doi.org/10.1016/j.coi.2010.02.012.

31. Sok, D., and Burton, D.R. (2018). Recent progress in broadly neutralizing antibodies to HIV. Nat. Immunol. *19*, 1179–1188. https://doi.org/10.1038/s41590-018-0235-7.

32. Yoon, H., Macke, J., West, A.P., Jr., Foley, B., Bjorkman, P.J., Korber, B., and Yusim, K. (2015). CATNAP: a tool to compile, analyze and tally neutralizing antibody panels. Nucleic Acids Res. *43*, W213–W219. https://doi.org/10.1093/nar/gkv404.

33. Foglierini, M., Nortier, P., Schelling, R., Winiger, R.R., Jacquet, P., O'Dell, S., Demurtas, D., Mpina, M., Lweno, O., Muller, Y.D., et al. (2024). RAIN: machine learning-based identification for HIV-1 bNAbs. Nat. Commun. *15*, 5339. https://doi.org/10.1038/s41467-024-49676-1.

34. Croce, D., Castellucci, G., and Basili, R. (2020). GAN-BERT: generative adversarial learning for robust text classification with a bunch of labeled examples. In Proceedings of the 58th Annual Meeting of the Association for Computational Linguistics, pp. 2114–2119. https://doi.org/10.18653/v1/2020.acl-main.191.

35. Burkovitz, A., and Ofran, Y. (2016). Understanding differences between synthetic and natural antibodies can help improve antibody engineering. mAbs *8*, 278–287. https://doi.org/10.1080/19420862.2015.1123365.

36. Soto, C., Bombardi, R.G., Branchizio, A., Kose, N., Matta, P., Sevy, A.M., Sinkovits, R.S., Gilchuk, P., Finn, J.A., and Crowe, J.E., Jr. (2019). High frequency of shared clonotypes in human B cell receptor repertoires. Nature *566*, 398–402. https://doi.org/10.1038/s41586-019-0934-8.

37. Regep, C., Georges, G., Shi, J., Popovic, B., and Deane, C.M. (2017). The H3 loop of antibodies shows unique structural characteristics. Proteins *85*, 1311–1318. https://doi.org/10.1002/prot.25291.

38. Jeliazkov, J.R., Sljoka, A., Kuroda, D., Tsuchimura, N., Katoh, N., Tsumoto, K., and Gray, J.J. (2018). Repertoire analysis of antibody CDR-H3 loops suggests affinity maturation does not typically result in rigidification. Front. Immunol. *9*, 413. https://doi.org/10.3389/fimmu.2018.00413.

39. Titanji, K., De Milito, A., Cagigi, A., Thorstensson, R., Grützmeier, S., Atlas, A., Hejdeman, B., Kroon, F.P., Lopalco, L., Nilsson, A., and Chiodi, F. (2006). Loss of memory B cells impairs maintenance of long-term serologic memory during HIV-1 infection. Blood *108*, 1580–1587. https://doi.org/10.1182/blood-2005-11-013383.

40. De Milito, A., Mörch, C., Sönnerborg, A., and Chiodi, F. (2001). Loss of memory (CD27) B lymphocytes in HIV-1 infection. Aids *15*, 957–964. https://doi.org/10.1097/00002030-200105250-00003.

41. Chong, Y., Ikematsu, H., Kikuchi, K., Yamamoto, M., Murata, M., Nishimura, M., Nabeshima, S., Kashiwagi, S., and Hayashi, J. (2004). Selective CD27 (memory) B cell reduction and characteristic B cell alteration in drug-naive and HAART-treated HIV type 1-infected patients. AIDS Res. Hum. Retroviruses *20*, 219–226. https://doi.org/10.1089/088922204773004941.

42. Nabi, R., Moldoveanu, Z., Wei, Q., Golub, E.T., Durkin, H.G., Greenblatt, R.M., Herold, B.C., Nowicki, M.J., Kassaye, S., Cho, M.W., et al. (2017). Differences in serum IgA responses to HIV-1 gp41 in elite controllers compared to viral suppressors on highly active antiretroviral therapy. PLoS One *12*, e0180245. https://doi.org/10.1371/journal.pone.0180245.

43. Ghraichy, M., Von Niederhäusern, V., Kovaltsuk, A., Galson, J.D., Deane, C.M., and Trück, J. (2021). Different B cell subpopulations show distinct patterns in their IgH repertoire metrics. eLife *10*, e73111. https://doi.org/10.7554/eLife.73111.

44. Hauser, M., Steinegger, M., and Söding, J. (2016). MMseqs software suite for fast and deep clustering and searching of large protein sequence sets. Bioinformatics *32*, 1323–1330. https://doi.org/10.1093/bioinformatics/btw006.

45. Soto, C., Finn, J.A., Willis, J.R., Day, S.B., Sinkovits, R.S., Jones, T., Schmitz, S., Meiler, J., Branchizio, A., and Crowe, J.E., Jr. (2020). PyIR: a scalable wrapper for processing billions of immunoglobulin and T cell receptor sequences using IgBLAST. BMC Bioinf. *21*, 314. https://doi.org/10.1186/s12859-020-03649-5.

46. Tsuruta, H., Yamazaki, H., Maeda, R., Tamura, R., Wei, J., Mariet, Z.E., Phloyphisut, P., Shimokawa, H., Ledsam, J.R., Colwell, L., and Imura, A. (2023). AVIDa-hIL6: a large-scale VHH dataset produced from an immunized alpaca for predicting antigen-antibody interactions. Advances in Neural Information Processing Systems *15*, 42077–42096.

47. Tsuruta, H., Yamazaki, H., Maeda, R., Tamura, R., and Imura, A. (2024). A SARS-CoV-2 interaction dataset and VHH sequence corpus for antibody language models. Advances in Neural Information Processing Systems *16*, 116149–116171.

48. Salazar, J., Liang, D., Nguyen, T.Q., and Kirchhoff, K. (2020). Masked language model scoring. In Proceedings of the 58th Annual Meeting of the Association for Computational Linguistics, pp. 2699–2712. https://doi.org/10.18653/v1/2020.acl-main.240.

49. Rodella, C. (2025). H3berta: A cdr-h3 specific language model for antibody repertoire analysis. Zenodo. doi:https://doi.org/10.5281/zenodo.17505849.

Patterns, Volume 7

Supplemental information

H3BERTa: A CDR-H3-specific

language model for antibody repertoire analysis

Chiara Rodella and Thomas Lemmin

Supplementary Information

A

Isotype	n seq.	Mean MLM	Perplexity
IgG	19	1.42	4.14
IgA	90	2.10	8.15
IgE	53	1.38	3.98
IgM	54	1.67	5.30
IgD	46	1.45	4.27

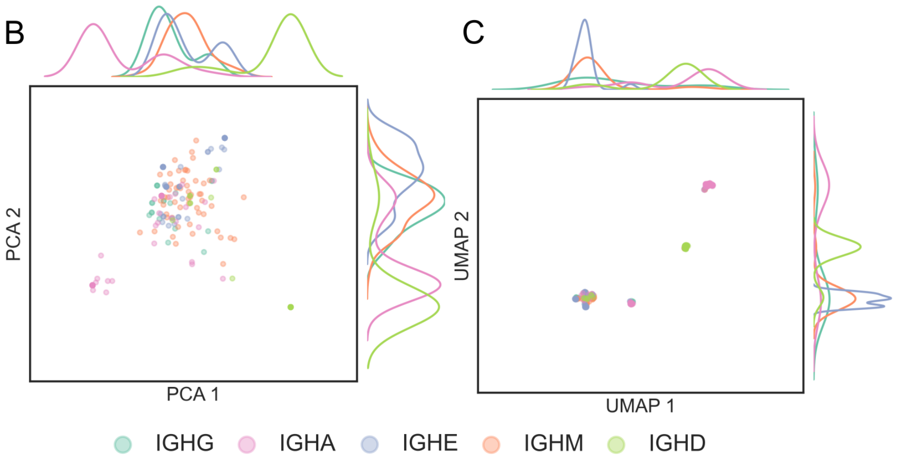

Figure S1: **H3BERTa performance and embedding structure across antibody isotypes.** (A) Mean masked language modeling (MLM) loss and perplexity for CDR-H3 sequences stratified by antibody isotype. (B) Principal component analysis (PCA) and (C) uniform manifold approximation and projection (UMAP) of H3BERTa-derived CDR-H3 embeddings, colored by isotype: IgG (light blue), IgA (pink), IgE (dark blue), IgM (red), and IgD (green). Each point represents a unique heavy-chain CDR-H3 sequence.

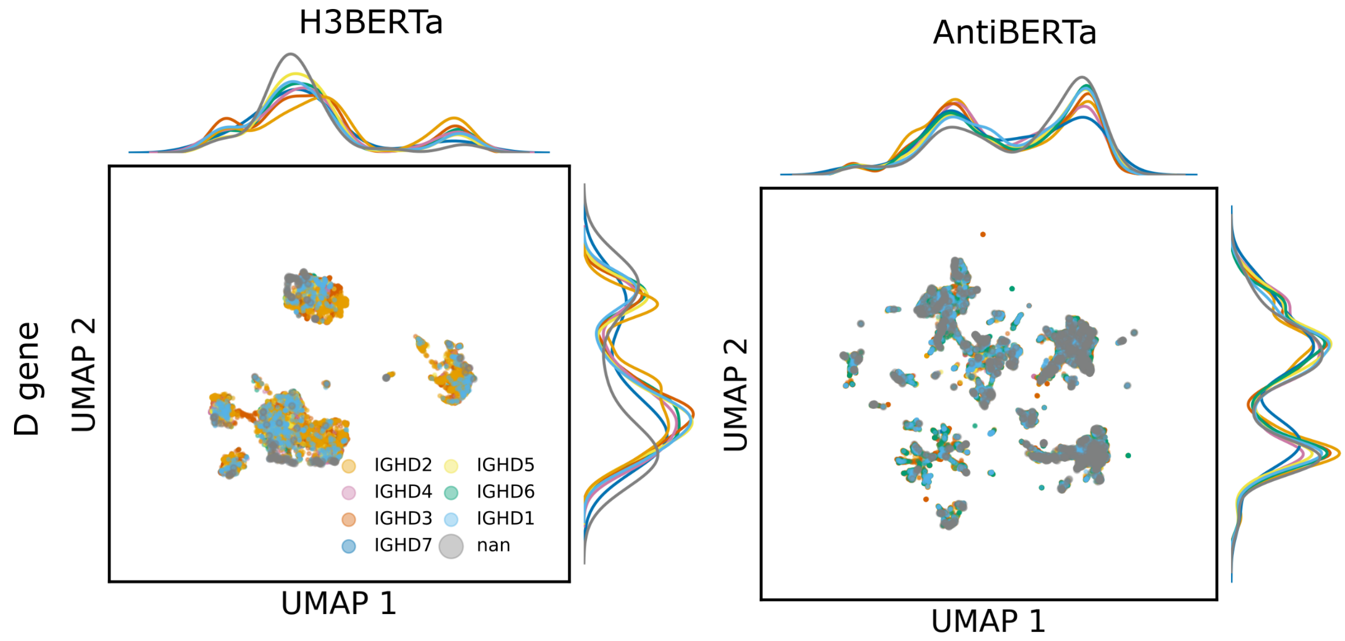

Figure S2: **UMAP visualization of H3BERTa and AntiBERTa-derived embeddings for CDR-H3 loops.** Each point represents a single CDR-H3 embedding colored by IGHD gene-segment usage. Marginal ridge plots alongside each UMAP axis depict the density of individual D-gene families.

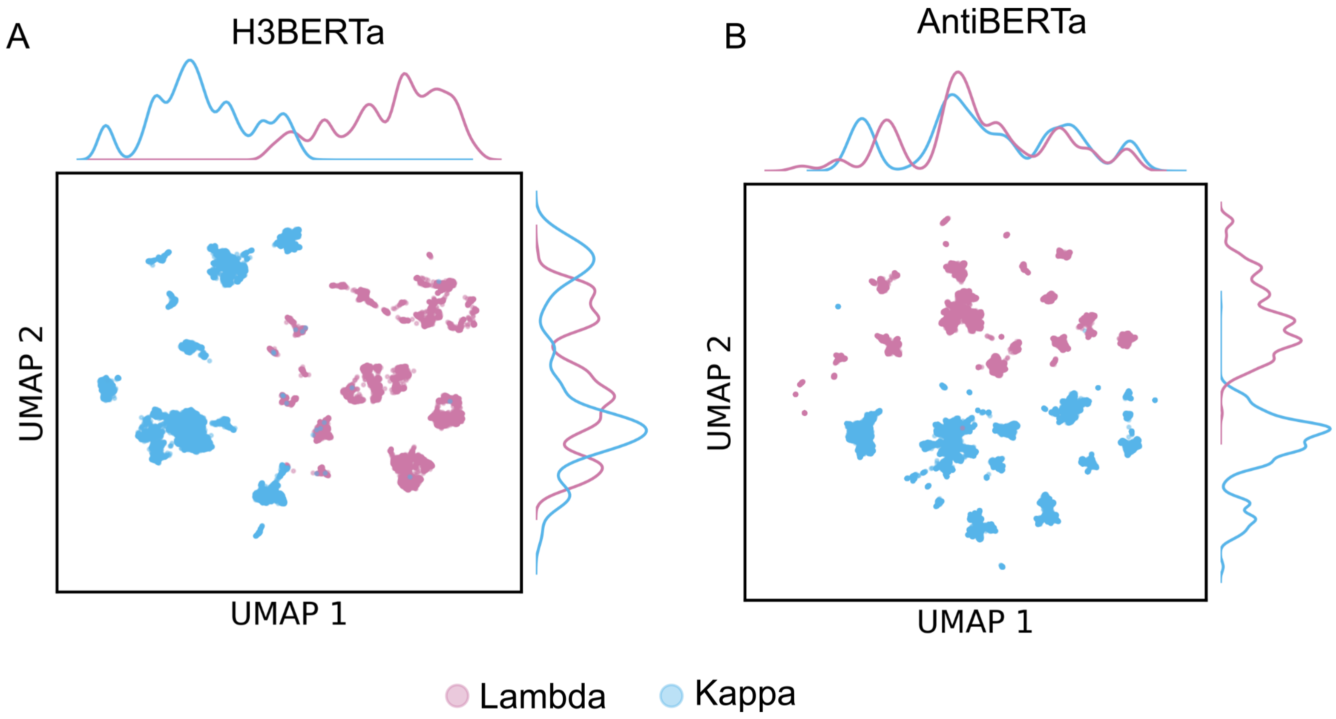

Figure S3: **Visualization of light-chain CDR3 embeddings.** Uniform manifold approximation and projection (UMAP) of CDR-L3 embeddings generated by H3BERTa (A) and full-length embeddings generated by AntiBERTa2 (B). Each point represents a unique antibody sequence and is colored by light-chain locus: lambda (pink) and kappa (blue).

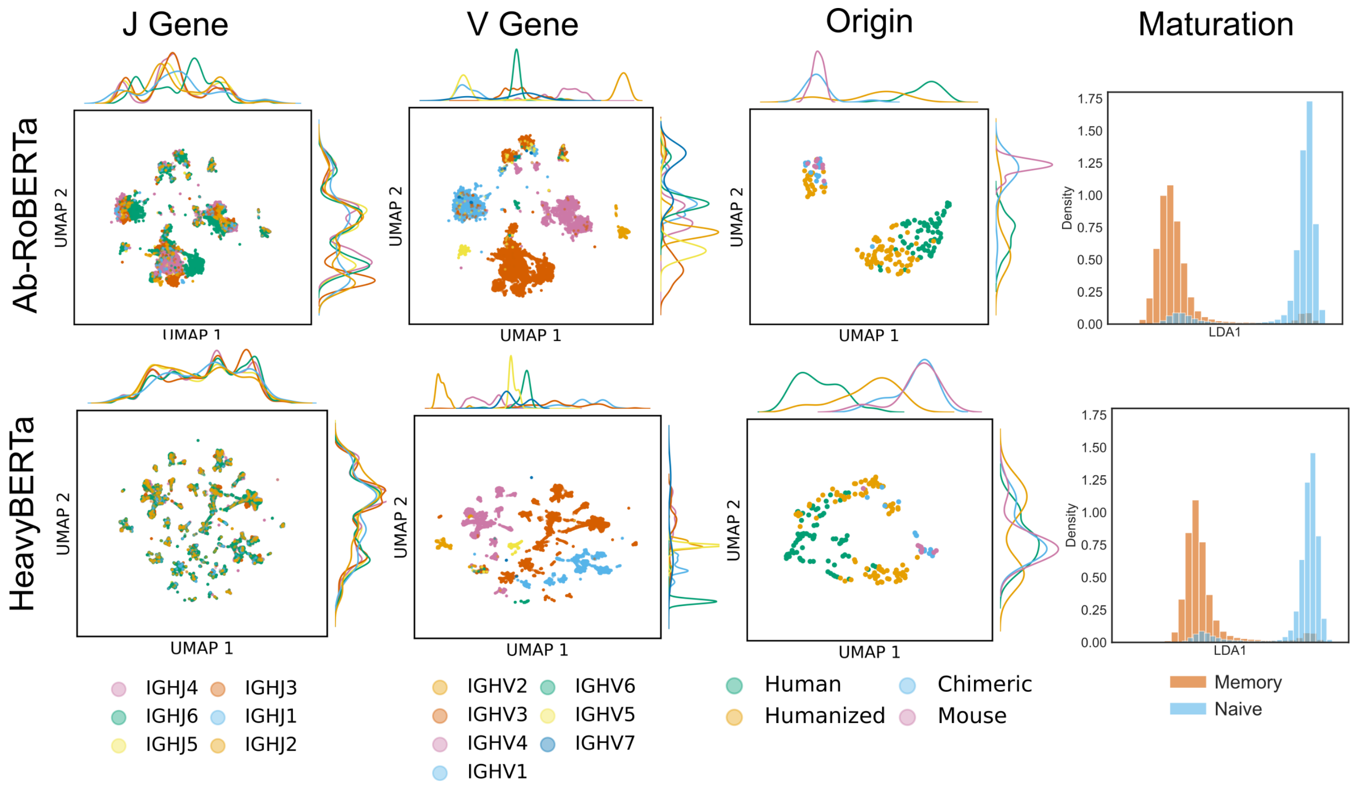

Figure S4: **Sequence embeddings generated by Ab-RoBERTa (top row) and HeavyBERTa (bottom row), visualized using UMAP or Linear Discriminant Analysis (LDA).** For UMAP panels, marginal distributions along each axis are shown as kernel density ridges. Each point represents a unique antibody heavy-chain sequence. UMAP projection of embeddings, colored by (A) IGHJ gene segment and (B) IGHV gene segment. (C) UMAP projection of embeddings from therapeutic antibodies, colored by annotated species origin: Human (green), Humanized (orange), Chimeric (light blue), and Mouse (pink). (D) LDA projection of embeddings from naive (blue) and memory (orange) B-cell sequences.

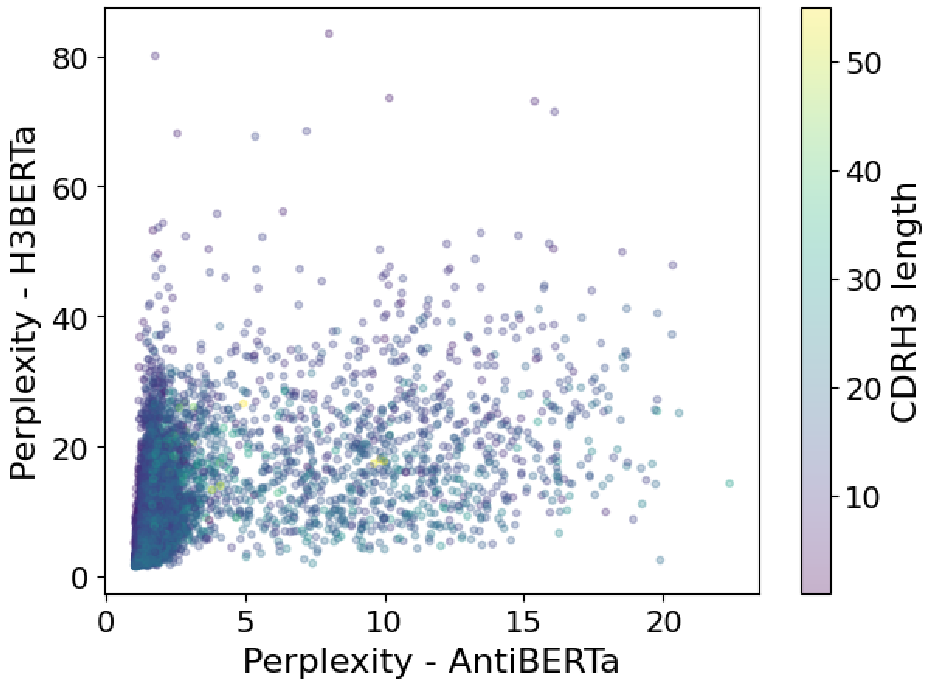

Figure S5: **Comparison of perplexity values calculated with AntiBERTa2 (x-axis) and H3BERTa (y-axis) on a healthy repertoire, specifically on the heavy chain and CDR-H3 loop, respectively.** Each point represents a unique CDR-H3 sequence: shifts to the right indicate an increase in perplexity for AntiBERTa2, and shifts upwards indicate an increase for H3BERTa. Points are colored according to CDR-H3 length using a continuous colormap.

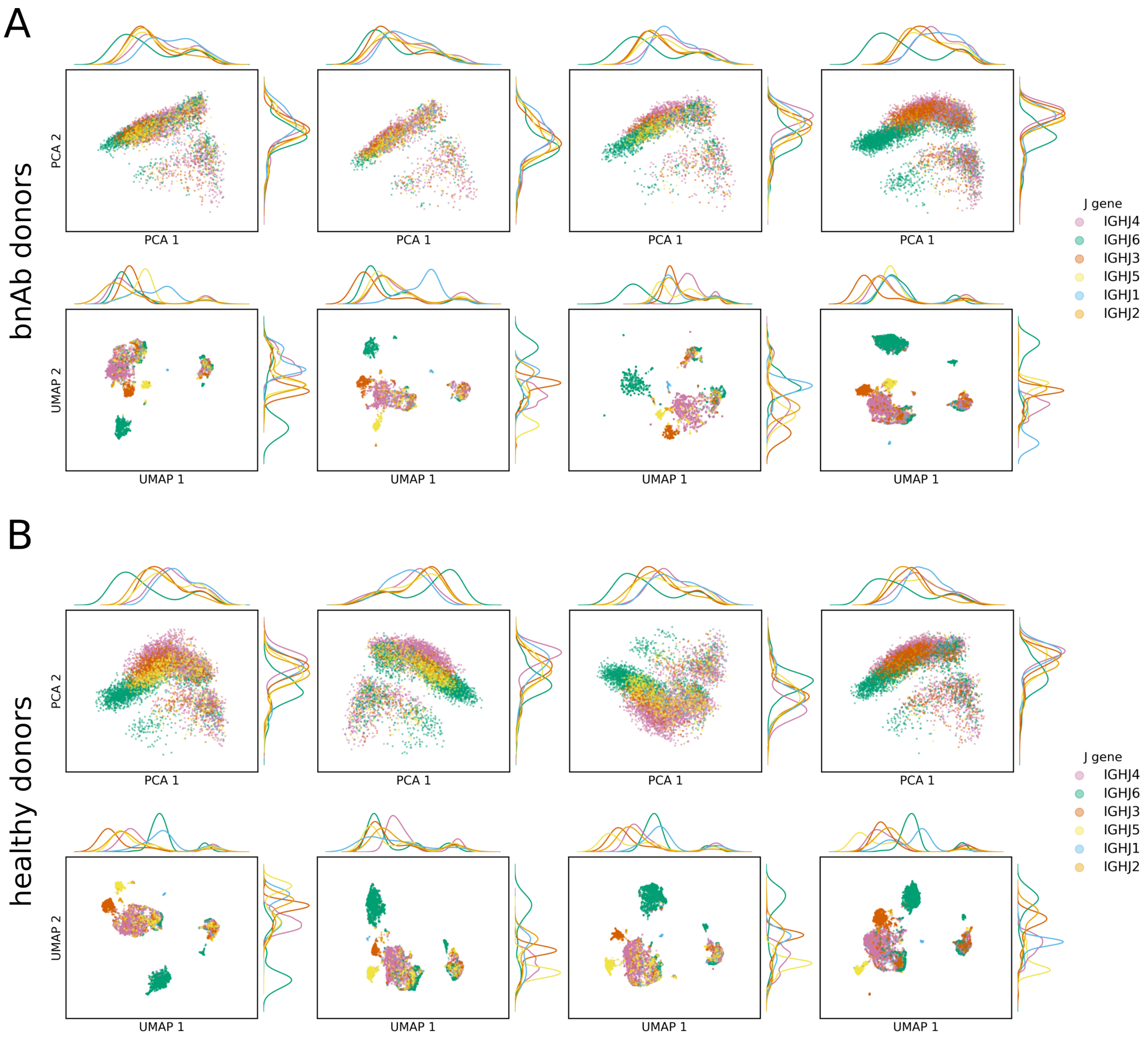

Figure S6: **Visualization of H3BERTa-derived embeddings for CDR-H3 loops from (A) bnAb donors and (B) healthy donors.** Each point represents a single CDR-H3 embedding colored by by IGHJ gene-segment usage. Marginal ridge plots alongside each PCA and UMAP axis depict the density of individual J-gene families.

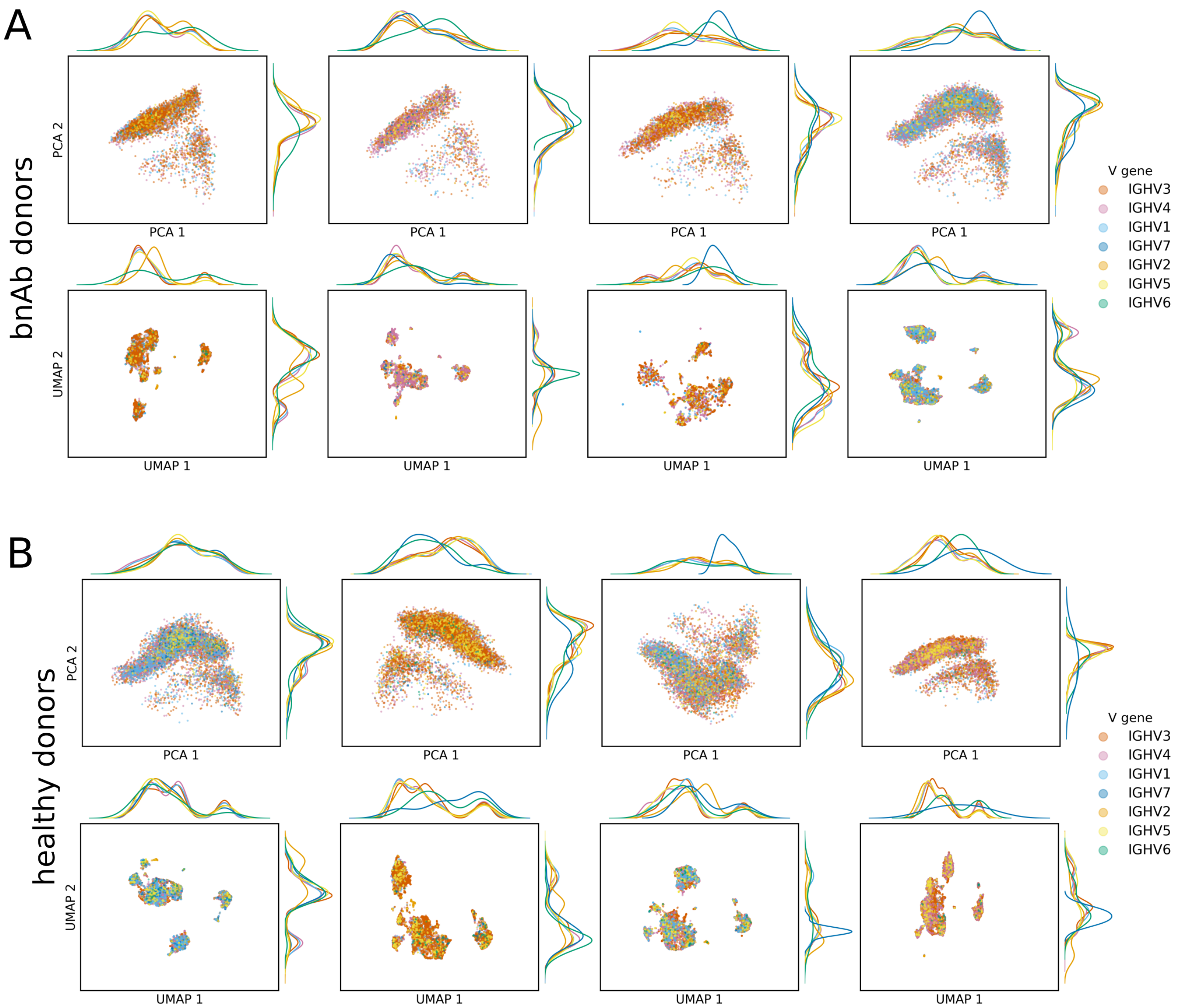

Figure S7: **Visualization of H3BERTa-derived embeddings for CDR-H3 loops from (A) bnAb donors and (B) healthy donors.** Each point represents a single CDR-H3 embedding colored by by IGHV gene-segment usage. Marginal ridge plots alongside each PCA and UMAP axis depict the density of individual V-gene families.

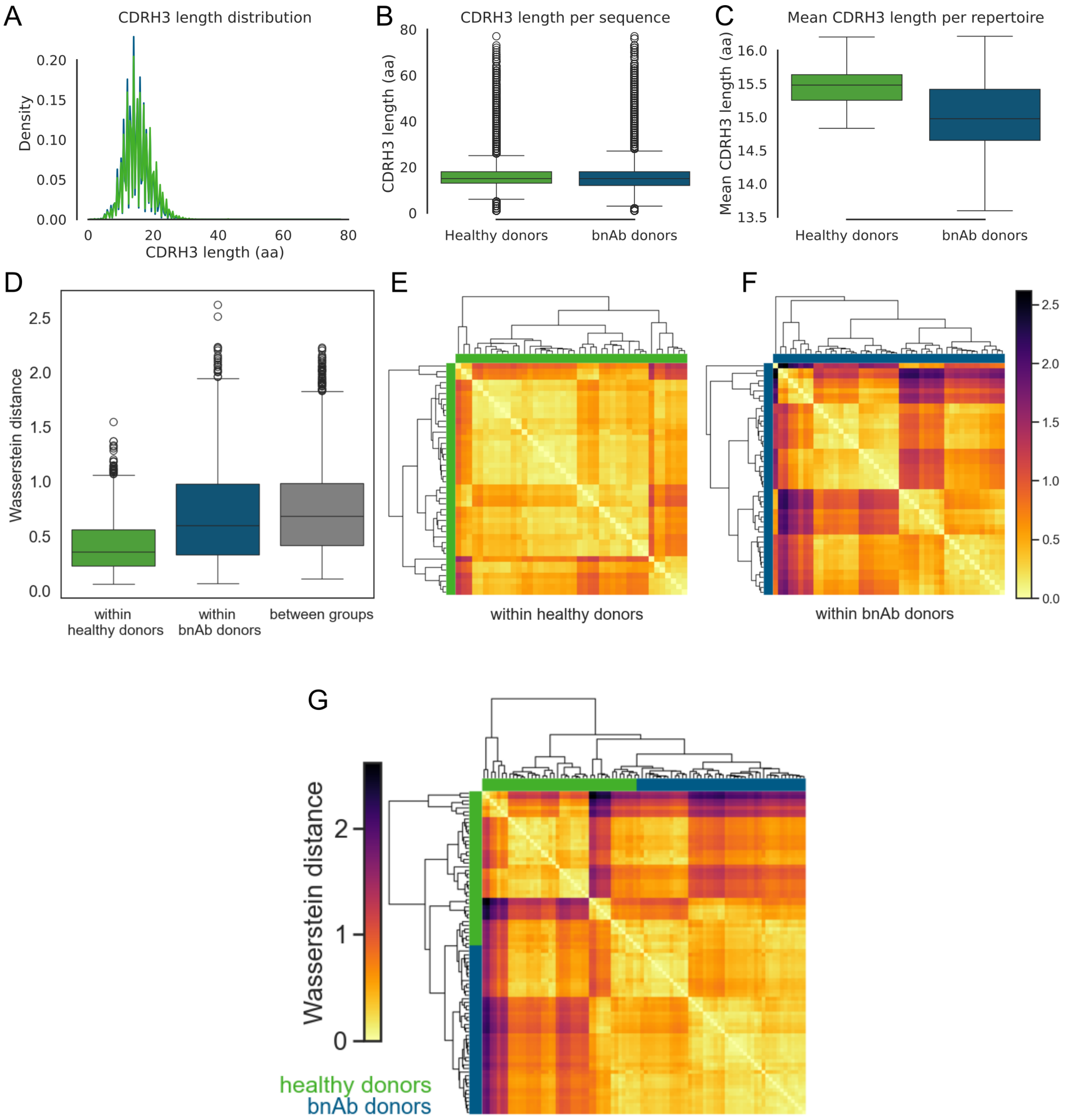

Figure S8: **Characterization of CDR-H3 length distributions.** (A) Kernel density estimate of CDR-H3 length distributions for healthy donors (green) and bnAb patients (blue). (B) Box plot of individual CDR-H3 sequence lengths; boxes indicate mean $\pm$ standard deviation, and outliers are shown as individual points. (C) Box plot of the average CDR-H3 length per repertoire for healthy and bnAb donors. (D) Box plot of Wasserstein distances between CDR-H3 length distributions across repertoires. (E-G) Ward clustering based on Wasserstein distances of CDR-H3 length distributions: (E) healthy donors, (F) bnAb donors, and (G) combined healthy and bnAb donors.

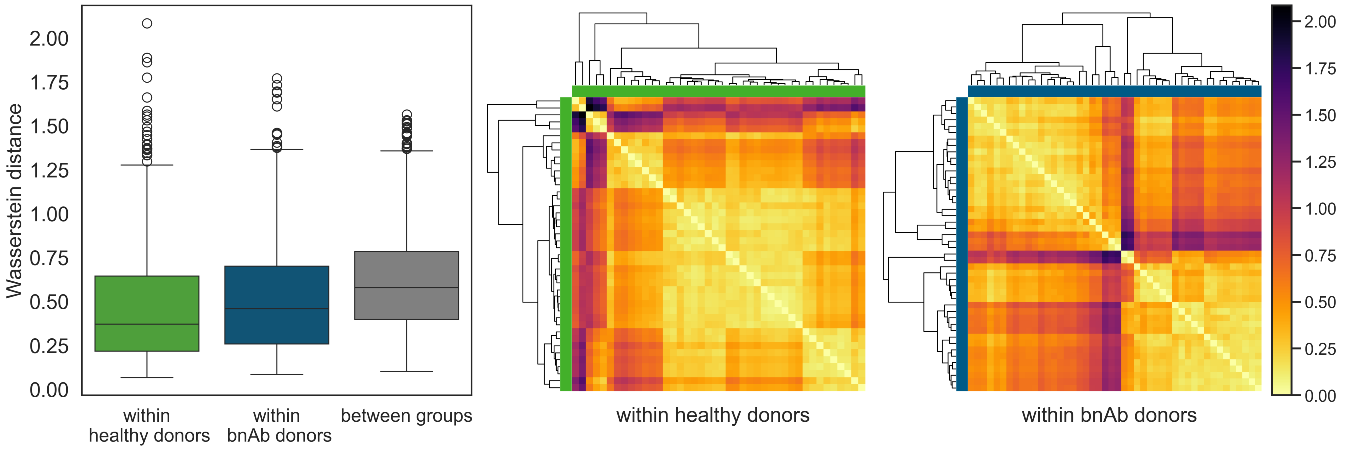

Figure S9: **Wasserstein distances analysis**(A) Box plots of pairwise Wasserstein distances between CDR-H3 perplexity distributions from H3BERTA. Distances are shown for comparisons within healthy donors (green), within bNAb donors (blue), and between the two cohorts (grey). Lower values indicate more similar repertoires. Healthy donors display the lowest median distance, whereas inter-cohort comparisons are shifted upward. (B) Heatmaps of the same pairwise distances. The left panel includes only healthy donors; the right panel includes only bNAb donors. Samples are ordered by hierarchical clustering to highlight similarity patterns. Colours progress from yellow (0; high similarity) through orange to black (= 2; high dissimilarity).

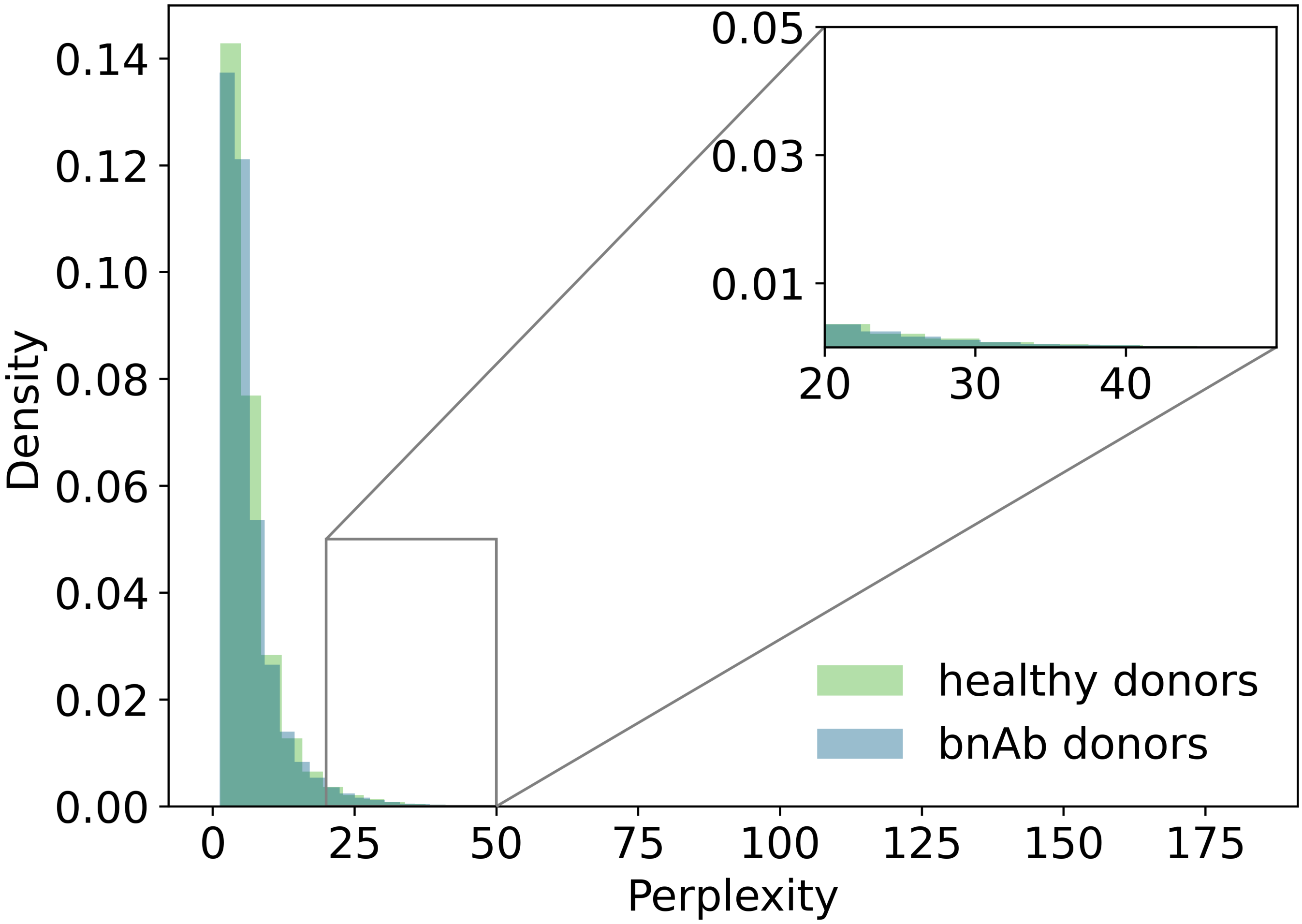

Figure S10: **Perplexity distributions of CDR-H3 sequences from healthy and bnAb donors.** Kernel density estimates show that both distributions are heavily right-skewed, with most sequences having low perplexity scores. An inset highlights the right tail of the distribution, where rare high-perplexity sequences are more visible.

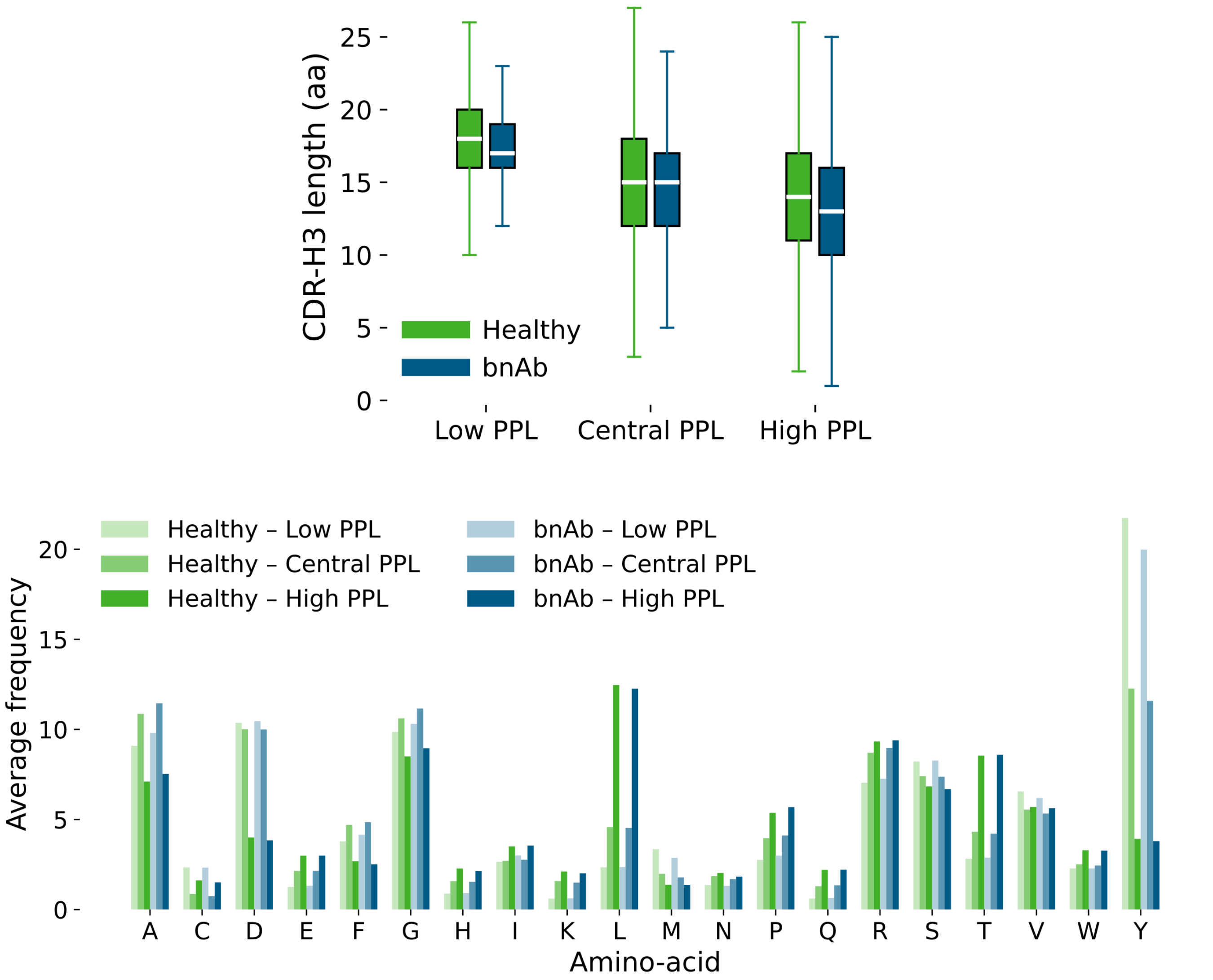

Figure S11: **Sequence features of CDR-H3 stratified by pseudo-perplexity (PPL) in healthy and bnAb donors.** Top: Box plots of CDR-H3 lengths for low-, central-, and high-PPL sequences, shown separately for healthy donors (green) and bnAb donors (blue). Bottom: Mean amino acid frequencies within each PPL bin, displayed for healthy donors (shades of green) and bnAb donors (shades of blue).

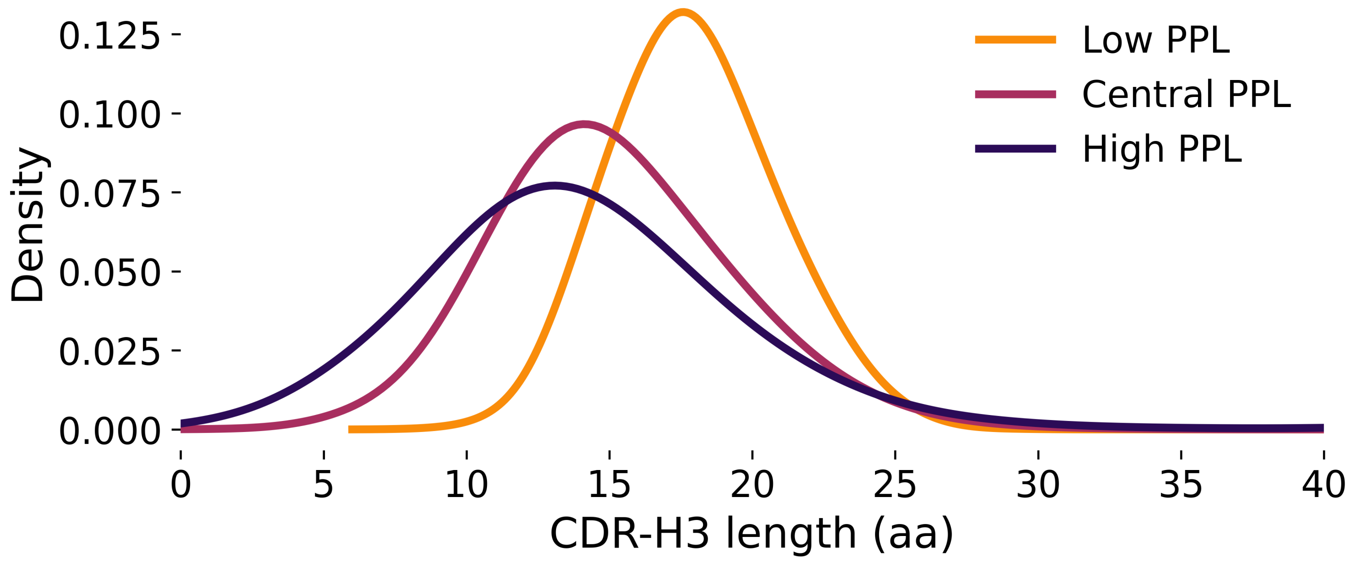

Figure S12: **Length distribution of CDR-H3 sequences across perplexity-defined groups.** Kernel density estimates of CDR-H3 amino acid lengths for sequences categorized into Low PPL (orange), Central PPL (magenta), and High PPL (dark purple) groups. While all groups display a peak in the 12-18 amino acid range, Low PPL sequences tend to be longer, indicating a potential association between lower model perplexity and naive CDR-H3 regions. CDR-H3 sequences with lengths greater than 40 amino acids were removed from the plot as outliers to improve the clarity of the density distribution.

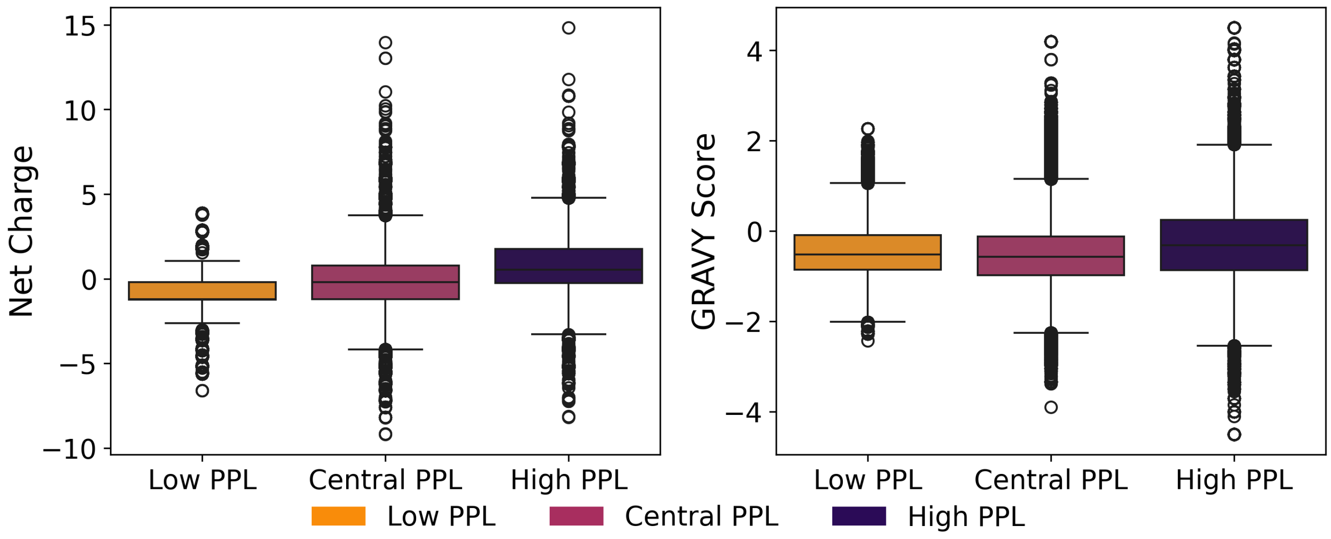

Figure S13: **Biochemical properties of CDR-H3 sequences across perplexity-defined groups.** Boxplots of (A) net charge at physiological pH (7.0) and (B) hydrophobicity (GRAVY score) for CDR-H3 sequences stratified by model-derived perplexity into Low, Central, and High PPL groups. Sequences in the Low PPL group exhibit significantly lower net charge compared to Central and High PPL groups (all $p < 0.001$, Mann-Whitney U test), suggesting a possible association between low perplexity and reduced electrostatic diversity. In contrast, GRAVY scores are relatively stable across groups, with only minor shifts despite statistically significant differences, indicating that overall hydrophobicity is not strongly differentiated by perplexity. Color-coded legends indicate PPL groups.

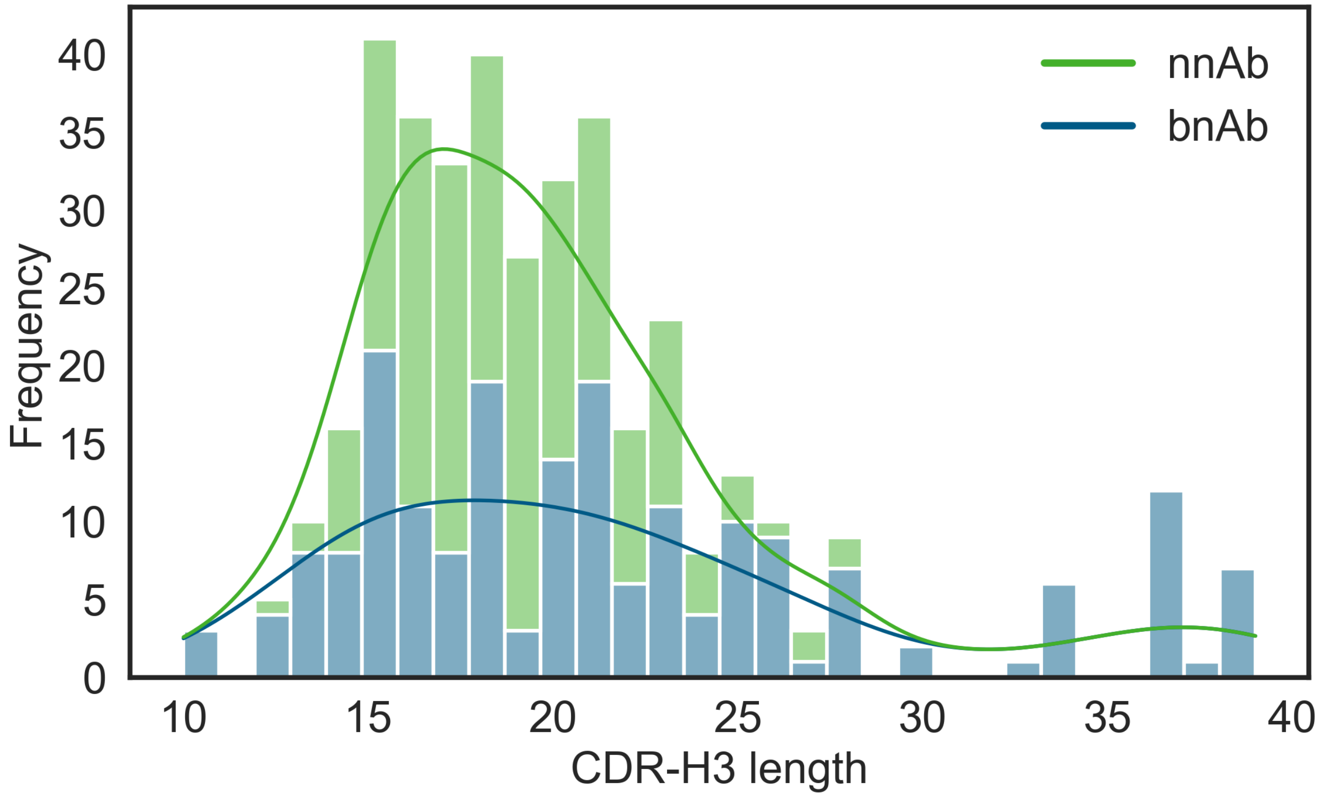

Figure S14: **Length distribution of CDR-H3 loops in bnAb and nnAb repertoires.** Histogram and kernel density estimates (KDE) of CDR-H3 amino acid lengths for broadly neutralizing antibodies (bnAbs, blue) and non-neutralizing antibodies (nnAbs, green). While both distributions peak in the 14-18 residue range, bnAbs tend to exhibit a broader and slightly right-shifted distribution, with a higher proportion of longer CDR-H3 loops compared to nnAbs.

Table S1: Mann–Whitney U test results for Net Charge at pH 7 and Hydrophobicity (GRAVY) across PPL groups.

**Comparison**	**Property**	**U statistic**	**p-value**
Low PPL vs Central PPL	Net Charge @ pH 7	1.26×10^{12}	$p < 0.001$
	Hydrophobicity (GRAVY)	1.69×10^{12}	$p < 0.001$
Low PPL vs High PPL	Net Charge @ pH 7	4.71×10^{10}	$p < 0.001$
	Hydrophobicity (GRAVY)	5.09×10^{10}	$p < 0.001$
Central PPL vs High PPL	Net Charge @ pH 7	1.16×10^{12}	$p < 0.001$
	Hydrophobicity (GRAVY)	8.12×10^{11}	$p < 0.001$

Table S2: SVM performance on validation and test sets. Metrics are reported overall and per class. Acc. = Accuracy, P = Precision, R = Recall, F1 = F1-score.

**Set**	**Acc.**	**bnAbs**			**nnAbs**		
		P	R	F1	P	R	F1
Validation	92.0%	1.00	0.83	0.91	0.86	1.00	0.92
Test	93.0%	0.95	0.90	0.92	0.90	0.95	0.93

Table S3: GAN-H3BERTa performance on validation and test sets. Metrics are reported overall and per class. Acc. = Accuracy, MCC = Matthews correlation coefficient, AUC = Area under the ROC curve, P = Precision, R = Recall, F1 = F1-score.

Set	Acc.	MCC	AUC	bnAbs			nnAbs		
				P	R	F1	P	R	F1
Validation	83.3%	0.67	0.83	0.80	0.89	0.84	0.88	0.78	0.82
Test	85.0%	0.71	0.85	0.79	0.95	0.86	0.94	0.75	0.83

Table S4: GAN-H3BERTa seed sensitivity analysis. Metrics are reported per seed on validation (val) and test sets, as computed with sklearn macro averages.

Seed	Val Accuracy	Val F1	Test Accuracy	Test F1
Model 1	0.81	0.80	0.82	0.82
Model 2	0.94	0.94	0.82	0.82
Model 3	0.81	0.80	0.85	0.85
Mean	0.85	0.85	0.83	0.83
Std	0.06	0.07	0.02	0.02
